# Oxylipin signaling in salt-stressed soybean is modulated by ligand-dependent interaction of Class II acyl-CoA-binding proteins with lipoxygenase

**DOI:** 10.1093/plcell/koab306

**Published:** 2021-12-17

**Authors:** Shiu-Cheung Lung, Sze Han Lai, Haiyang Wang, Xiuying Zhang, Ailin Liu, Ze-Hua Guo, Hon-Ming Lam, Mee-Len Chye

**Affiliations:** 1 School of Biological Sciences, The University of Hong Kong, Pokfulam, Hong Kong, China; 2 School of Life Sciences and Centre for Soybean Research of the State Key Laboratory of Agrobiotechnology, The Chinese University of Hong Kong, Shatin, Hong Kong, China

## Abstract

Plant lipoxygenases (LOXs) oxygenate linoleic and linolenic acids, creating hydroperoxy derivatives, and from these, jasmonates and other oxylipins are derived. Despite the importance of oxylipin signaling, its activation mechanism remains largely unknown. Here, we show that soybean ACYL-COA-BINDING PROTEIN3 (ACBP3) and ACBP4, two Class II acyl-CoA-binding proteins, suppressed activity of the vegetative LOX homolog VLXB by sequestering it at the endoplasmic reticulum. The ACBP4–VLXB interaction was facilitated by linoleoyl-CoA and linolenoyl-CoA, which competed with phosphatidic acid (PA) for ACBP4 binding. In salt-stressed roots, alternative splicing produced ACBP variants incapable of VLXB interaction. Overexpression of the variants enhanced LOX activity and salt tolerance in Arabidopsis and soybean hairy roots, whereas overexpressors of the native forms exhibited reciprocal phenotypes. Consistently, the differential alternative splicing pattern in two soybean genotypes coincided with their difference in salt-induced lipid peroxidation. Salt-treated soybean roots were enriched in C32:0-PA species that showed high affinity to Class II ACBPs. We conclude that PA signaling and alternative splicing suppress ligand-dependent interaction of Class II ACBPs with VLXB, thereby triggering lipid peroxidation during salt stress. Hence, our findings unveil a dual mechanism that initiates the onset of oxylipin signaling in the salinity response.

## Teaser

Phosphatidic acid signaling and alternative splicing inhibit ligand-dependent interaction of Class II acyl-CoA-binding proteins with lipoxygenase, triggering oxylipin signaling in salt-stressed soybean.

##  

**Figure:**
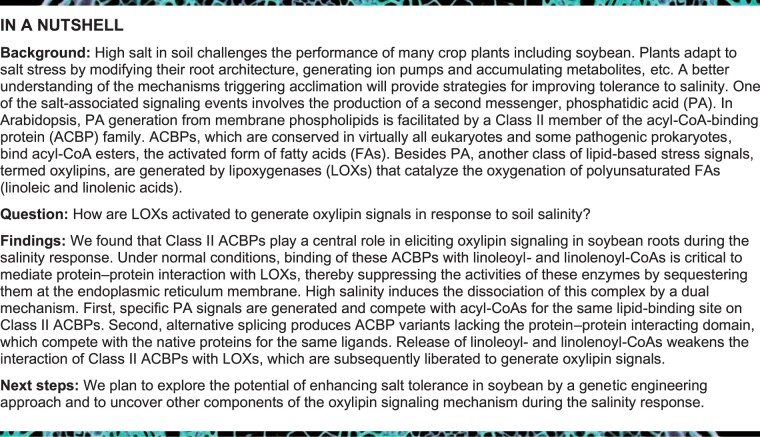


## Introduction

Plant lipoxygenases (LOXs) catalyze dioxygenation of linoleic (C18:2) and linolenic (C18:3) acids to form the corresponding hydroperoxy fatty acids (FAs), which are used for oxylipin synthesis (Andreou and Feussner, 2009; Wasternack and Feussner, 2018). Jasmonates (JAs), which are among the best studied oxylipins, are derived from 13-hydroperoxides via the plastidial type-2 LOX pathway (Wasternack and Song, 2017). In contrast, 9-hydroperoxides are generated by type-1 LOXs, which have been characterized predominantly in cultivated soybean (*Glycine max*) due to their high expression (Grayburn et al., 1991; Kato et al., 1992). In earlier work, the extraplastidal localization of type-1 GmLOXs supported their role in lipid metabolism (Stephenson et al., 1998; Fischer et al., 1999; Turner et al., 2011), and their signaling function is suggested by their stress-inducible expression (Bell and Mullet, 1991).

The elicitation of type-1 LOX-dependent peroxidative pathways is considered an innate response against microbial infection in Arabidopsis (*Arabidopsis thaliana*; Vellosillo et al., 2007; Lόpez et al., 2011; Vicente et al., 2012; Marcos et al., 2015), maize (*Zea mays*; Christensen et al., 2015), pepper (*Capsicum annuum*; Hwang and Hwang, 2010), potato (*Solanum tuberosum*; Göbel et al., 2002), and tobacco (*Nicotiana tabacum*; Rustérucci et al., 1999; Hamberg et al., 2003). This innate response is primarily for the generation of FA hydroxides and hydroperoxides as defense signals. In higher plants, type-1 LOXs are also crucial for mediating responses to adversities such as wounding (Nalam et al., 2012; Zhou et al., 2014), oxidative damage (Lόpez et al., 2011; Keunen et al., 2013), drought, and salinity (Ben-Hayyim et al., 2001; Hou et al., 2015; Lim et al., 2015). Although the signaling roles of oxylipins in development and stress responses have drawn much attention (Wasternack and Song, 2017; Yu et al., 2020a), the relevant activation mechanism remains largely unknown.

The regulatory functions of acyl-CoA-binding (ACB) proteins (ACBPs) have been extensively studied in plant development and signaling (Du et al., 2016; Lung and Chye, 2016a, 2019). De novo synthesized FAs are activated, forming CoA-thioesters after their export from the plastids. The cytosolic acyl-CoA pool is maintained by Class I ACBPs, which play ubiquitous developmental roles (Meng et al., 2011). For example, *A.* *thaliana* AtACBP6 influences the composition of seed triacylglycerol (Guo et al., 2019a) and phloem oxylipins (Ye et al., 2016), and OsACBP2 affects rice grain size and bran oil content (Guo et al., 2019b). Besides Class I ACBPs, there are multidomain homologs, including ankyrin (ANK) -repeat-containing ACBPs (Class II), large ACBPs (Class III), and kelch-ACBPs (Class IV) (Meng et al., 2011). The four classes of plant ACBPs are functionally diversified by differences in ligand selectivity, protein–protein interaction network, and spatiotemporal and stress-responsive expression patterns (Lung and Chye, 2016b). The widespread organellar distribution of ACBPs is also crucial for intracellular trafficking of acyl-CoAs from the cytosolic pool to several subcellular destinations (Lung and Chye, 2016c). For instance, Class II AtACBP1 facilitates recruitment of cytosolic acyl-CoAs for chain extension at the endoplasmic reticulum (ER) to form very-long-chain precursors for cuticular wax synthesis (Xue et al., 2014).

Class II ACBPs uniquely possess an ANK domain that facilitates protein–protein interactions, and their functional specificity is intimately tied to their protein partners in Arabidopsis. At the plasma membrane, Class II AtACBP1 and AtACBP2 sequester an ethylene response transcription factor (RAP2.12), which is released into the nucleus for hypoxic gene activation under anaerobic conditions (Licausi et al., 2011; Schmidt et al., 2018). AtACBP2 also interacts with FARNESYLATED PROTEIN6 and LYSOPHOSPHOLIPASE2 (LYSOPL2) for adaptive responses to heavy metal stress (Gao et al., 2009, 2010). Moreover, the ER-localized AtACBP1 partners with sterol C4-methyl oxidases (SMO1-1 and SMO1-2) to control the synthesis of important signals for embryogenesis and cell fate determination (Lung et al., 2017, 2018). The interaction of AtACBP1 with PHOSPHOLIPASE Dα1 (PLDα1) was shown to facilitate the generation of PA signals for stress and hormonal responses (Du et al., 2010, 2013a). Whereas *atacbp1* seeds were less dormant, the higher dormancy of AtACBP1-overexpressing seeds was attributed to their abscisic acid (ABA) hypersensitivity (Du et al., 2013a). The ABA-related function of AtACBP1 was further substantiated by its interaction with ABA-RESPONSIVE ELEMENT BINDING PROTEIN1 (AREB1) (Chen et al., 2018). During seed germination and seedling establishment, AtACBP1 overexpressors were more sensitive than the control plants to salt and mannitol treatment (Chen et al., 2018). Not surprisingly, the *atacbp1* mutants were less sensitive to these treatments. AtACBP1 forms a membrane-associated docking site for tethering AREB1, which is liberated into the nucleus for activating ABA-responsive gene transcription during salinity and osmotic stresses (Chen et al., 2018).

Soil salinity poses hyperosmotic, hyperionic, and oxidative challenges on most plants (van Zelm et al., 2020). The progressive loss of soybean productivity at rising soil salinity has been documented by Phang et al. (2008), and the adaptability of cultivated soybean to environmental adversity has diminished after domestication from its wild progenitor (*Glycine soja*) (Lam et al., 2010). In contrast, wild soybean varieties exhibit higher genomic diversity, from which desirable alleles are useful for crop improvement (Lam et al., 2010). For instance, whole-genome sequencing of the wild W05 soybean identified a functional ion transporter conferring salt tolerance that is disrupted in the salt-sensitive cultivars (Qi et al., 2014). Plants naturally acclimate to soil salinity by adaptive changes (e.g. ion homeostasis maintenance, osmoregulation, metabolite accumulation, and root morphological plasticity), implicating genome-wide reprogramming of gene expression (Phang et al., 2008; van Zelm et al., 2020). Salt stress also induces alternative gene splicing (Feng et al., 2015). Such posttranscriptional regulation generates multiple transcripts from the same allele leading to proteome diversification, whose significance has been elucidated with some proteins (Feng et al., 2015; Laloum et al., 2018). However, the physiological relevance of others awaits further studies.

Here, we identified three splice variants of Class II GmACBPs in salt-stressed soybean roots and applied them successfully for engineering plant salt tolerance. This exemplifies both an adaptive response induced by alternative splicing and a new genetic strategy for crop improvement. Furthermore, our study revealed ligand-dependent interaction of Class II GmACBPs with a salt-inducible type-1 LOX at the ER. The suppression of this interaction by PA signaling and alternative splicing in response to salinity appears to be a dual mechanism to trigger the onset of oxylipin signaling.

## Results

### Class II ACBPs are alternatively spliced in salt-stressed soybean roots

**Figure 1:**
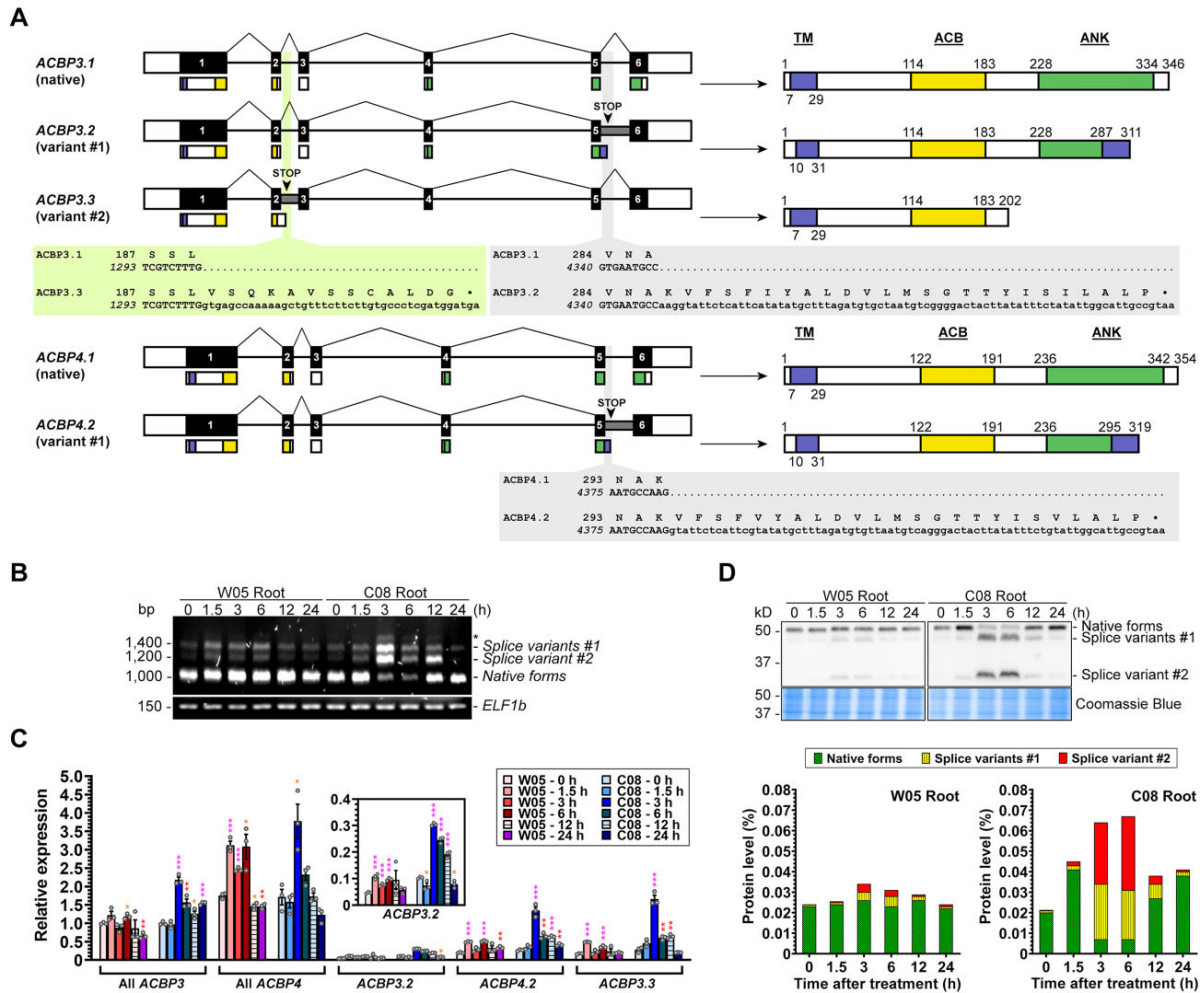
Alternative splicing of Class II *ACBP3* and *ACBP4* produces truncated proteins in soybean roots under high salinity. A, Schematic diagram showing alternative splicing of *ACBP3* and *ACBP4* with their translational products comprising the TM, ACB, and ANK-repeat domains. Exons (numbered boxes) with coding regions (black) and retained introns (gray) are displayed with corresponding translational product shown underneath. Callout boxes compare codons in splice variants (bottom) preceding a premature stop codon (asterisk) with the native peptide (top). B, RT-PCR analysis of Class II *ACBP* mRNAs in 3-week-old wild W05 and cultivated C08 soybean roots upon 0.9% (w/v) NaCl treatment. The primer pair used can amplify the entire ORF of both *ACBP3* and *ACBP4*. RT-PCR revealed the native forms (*ACBP3.1* and *ACBP4.1*), splice variants #1 with Intron V (*ACBP3.2* and *ACBP4.2*), splice variant #2 with Intron II (*ACBP3.3*), and a splice variant with both Intron V (as in *ACBP3.2*) and Intron II (as in *ACBP3.3*), as indicated by an asterisk. *ELF1b*, a stable reference gene for NaCl treatment (Yim et al., 2015), was used as a loading control. C, Quantitative RT-PCR analysis of Class II *ACBP* mRNAs in 3-week-old W05 and C08 roots upon 0.9% (w/v) NaCl treatment. Data were normalized against *ELF1b*. The values for “All *ACBP3*” at 0 h were arbitrarily set to 1. Each bar represents the mean ± sem from three independent experiments with different plants. Asterisks indicate statistically significant (*P < 0.05; **P < 0.01; ***P < 0.001) difference from 0 h by Student’s *t* test (Supplemental Data Set S1). D, Immunoblot analysis of ACBP3 and ACBP4 proteins in 3-week-old W05 and C08 roots upon 0.9% (w/v) NaCl treatment. Total proteins (25 μg/lane) were resolved on 12% SDS-PAGE and analyzed by Coomassie blue staining and immunoblotting using anti-ACBP3 antibodies to cross-react with ACBP3 and ACBP4 (upper panel). The native forms include 38-kD ACBP3.1 (apparent: 52 kD) and 39-kD ACBP4.1 (apparent: 52 kD); the splice variants #1 include 34-kD ACBP3.2 (apparent: 45 kD) and 35-kD ACBP4.2 (apparent: 45 kD); the splice variant #2 is 22-kD ACBP3.3 (apparent: 30 kD). Cross-reacting bands were quantified by densitometric analysis (lower panels). Protein levels were calculated from a calibration curve established using purified recombinant proteins.

Two of the 11 soybean ACBP isoforms (ACBP3 and ACBP4) belong to Class II (Azlan et al., 2021) and share 95% amino acid sequence homology (Supplemental Figure S1). Their coding sequences (Supplemental Figure S2) and exon–intron architectures (Figure 1, A) are well conserved. The nucleotide sequences of *ACBP4* are identical in wild W05 and cultivated C08 soybeans, that is, the two accessions used in this study (Supplemental Figure S2). *ACBP3* sequences exhibit a nonimpactful single-nucleotide polymorphism between W05 and C08, resulting in different codons for Asp-79 (Supplemental Figure S2). Thus, GmACBP3 and GmACBP4 as mentioned hereinafter represent identical protein counterparts from wild W05 soybean.

Analysis of Class II *ACBP* mRNAs revealed alternative splicing of *ACBP3* and *ACBP4* (Figure 1). Besides the native forms, designated as *ACBP3.1* and *ACBP4.1* (Figure 1, A), larger amplicons arose from Intron II and/or V retention (Figure 1, B). Intron V retention produced splice variants *ACBP3.2* and *ACBP4.2*, and Intron II retention generated variant *ACBP3.3* (Figure 1, A). The salt-tolerant W05 roots accumulated the splice variants in early response, that is, 1.5–6 h posttreatment (hpt), while these variants were even more abundant in salt-sensitive C08 roots after NaCl treatment (Figure 1, B). To differentially monitor alternative splicing of *ACBP3* and *ACBP4*, isoform-specific primers were designed for a time-course study using quantitative real-time (qRT)-PCR. In W05 roots, both splice variants of *ACBP3* showed the highest magnitude of increase (1.3- and 1.9-fold) at 1.5 hpt in comparison with untreated roots, while *ACBP4.2* peaked (1.6-fold) at 6 hpt (Figure 1, C). C08 roots produced the maximum level of all three splice variants (2.0–4.9-fold) at 3 hpt (Figure 1, C).

Next, the potential translation of the three splice variants into stable proteins was examined. A premature stop codon in the retained Intron V (*ACBP3.2* and *ACBP4.2*) and Intron II (*ACBP3.3*) would lead to partial and complete truncation of the ANK domain, respectively (Figure 1, A). The presence of truncated Class II ACBPs was confirmed by immunoblotting with anti-ACBP3 antibodies, which could cross-react with both ACBP3 and ACBP4 (Figure 1, D). In NaCl-treated W05 and C08 roots, native ACBP3.1 and ACBP4.1 (apparent: 52 kD) were detected with two smaller bands corresponding to ACBP3.2 and ACBP4.2 (apparent: 45 kD), and to ACBP3.3 (apparent: 30 kD) (Figure 1, D). In C08 roots, the truncated proteins dominated at 3 and 6 hpt (Figure 1, D), in correlation with their mRNA (Figure 1, B and C). C08 roots had a higher level of salt stress-induced truncated ACBP3 and ACBP4 accumulation at 3 and 6 hpt than W05 roots (Figure 1, D). Thus, it appears that alternative splicing of Class II *ACBPs* produced protein variants with truncated ANK domain in soybean roots in response to salinity.

### Class II GmACBPs sequester type-1 LOX at the ER

**Figure 2:**
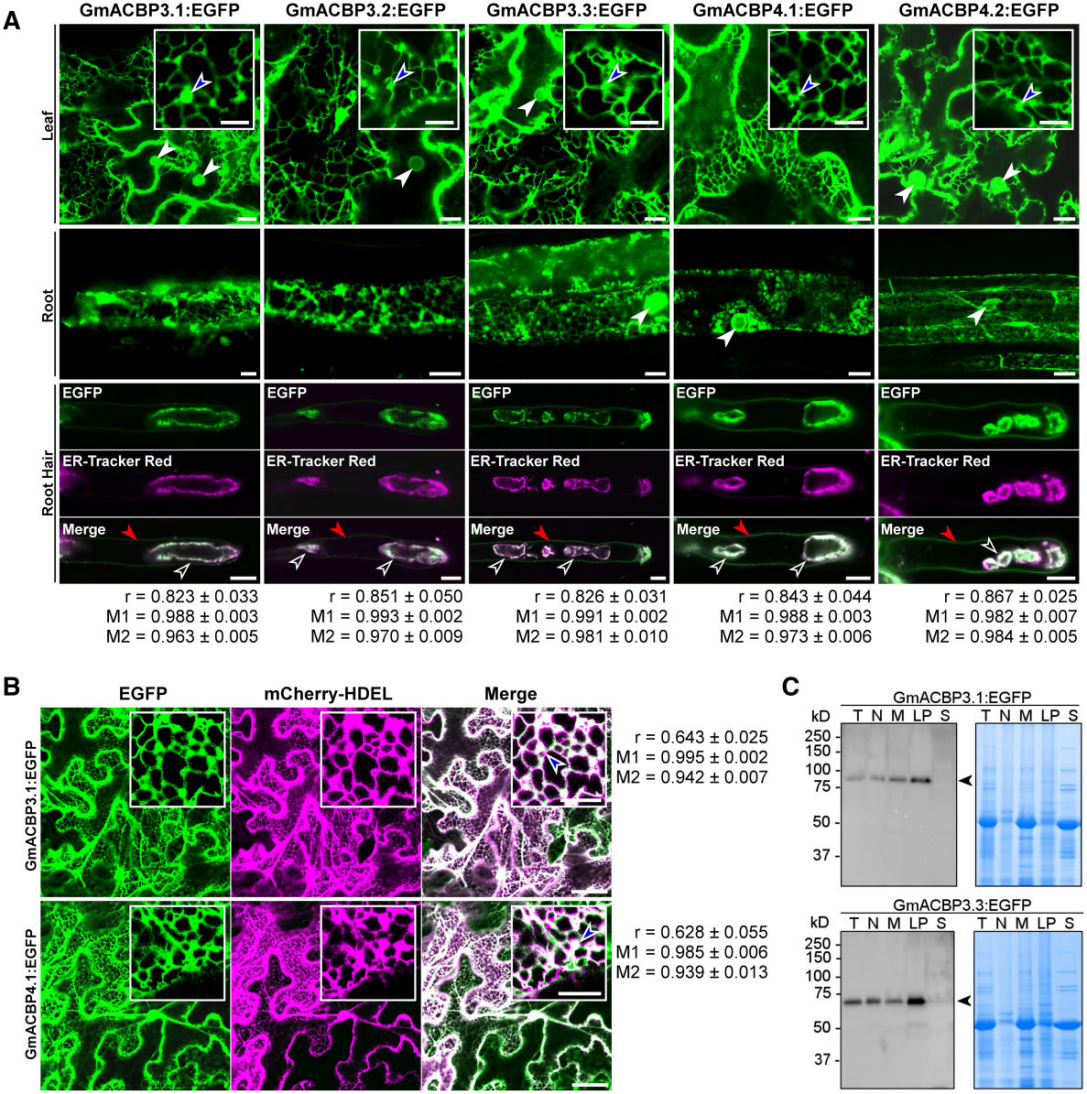
Native forms and splice variants of GmACBP3 and GmACBP4 are ER-localized. The ORF of native forms and splice variants of *GmACBP3* and *GmACBP4* was used to generate EGFP-fusion constructs for stable transformation of Arabidopsis and transient expression in *N. benthamiana*. Subcellular localization of EGFP-fusion proteins was examined by confocal laser scanning microscopy in (A–B) and subcellular fractionation in (C). A, In 3-week-old transgenic Arabidopsis, signals were detected at the perinuclear ER (solid arrowheads), ER cisternae (blue arrowheads), and ER tubules. In root hair cells of 7-day-old seedlings, EGFP-fusion proteins were colocalized with the ER-Tracker signals at the membrane of ER-derived vesicles (open arrowheads), despite additional EGFP signals at the plasma membrane (red arrowheads). Pearson’s correlation coefficient (*r*), Manders’ overlap coefficients M1 (fraction of EGFP overlapping ER-Tracker Red), and M2 (fraction of ER-Tracker Red overlapping EGFP) were computed from regions of interest of 400 × 100 pixels. Values represent the mean ± sem of 10 cells. Bars = 10 or 5 μm (insets). B, In agroinfiltrated *N. benthamiana* leaf epidermal cells, GmACBP3.1:EGFP and GmACBP4.1:EGFP were colocalized with the mCherry-HDEL ER marker at the ER cisternae (blue arrowheads) and ER tubules. Pearson’s correlation coefficient (*r*), Manders’ overlap coefficients M1 (fraction of EGFP overlapping mCherry-HDEL), and M2 (fraction of mCherry-HDEL overlapping EGFP) were computed from regions of interest of 150 × 150 pixels. Values represent the mean ± sem of 10 cells. Bars = 50 or 10 μm (insets). C, Subcellular fractionation of GmACBP3.1:EGFP and GmACBP3.3:EGFP in 10-day-old transgenic Arabidopsis shoots. Proteins (30 μg/lane) from total crude extracts (T), nuclei (N), membranes (M), large particles (LP), and soluble fractions (S) were resolved on 10% SDS-PAGE and analyzed by Coomassie blue staining and immunoblotting using anti-ACBP3 antibodies. Arrowheads indicate target bands of 65-kD GmACBP3.1:EGFP (apparent: 80 kD) and 49-kD GmACBP3.3:EGFP (apparent: 65 kD).

Confocal laser scanning microscopy revealed that EGFP-fusion proteins of the native (GmACBP3.1 and GmACBP4.1) and variant (GmACBP3.2, GmACBP3.3, and GmACBP4.2) forms of Class II GmACBPs were targeted to the ER structures, including the perinuclear ER, ER cisternae, and tubular ER network, in leaf epidermal and root cells of transgenic Arabidopsis (Figure 2, A). In root hair cells, all five EGFP-fusion proteins were colocalized with the ER-Tracker at the membrane of ER-derived vesicles, besides additional EGFP signals at the plasma membrane (Figure 2, A). GmACBP3.1:EGFP and GmACBP4.1:EGFP were also colocalized with the mCherry-HDEL marker at the ER cisternae and tubular ER network in *Nicotiana benthamiana* leaf epidermal cells (Figure 2, B). Colocalization of EGFP-fusion proteins with the ER-Tracker (Figure 2, A) and mCherry-HDEL marker (Figure 2, B) was confirmed by using the Pearson’s correlation coefficient (*r*) and Manders’ overlap coefficients (Bolte and Cordelières, 2006). Subcellular fractionation showed that the soluble fractions of crude protein extracts from transgenic Arabidopsis were devoid of GmACBP3.1:EGFP and GmACBP3.3:EGFP, indicating their membrane association (Figure 2, C). Thus, the C-terminal truncation in the splice variants of Class II GmACBPs did not alter their ER localization, in agreement with the N-terminal location of their transmembrane (TM) domain (Supplemental Figure S3, A).

**Figure 3:**
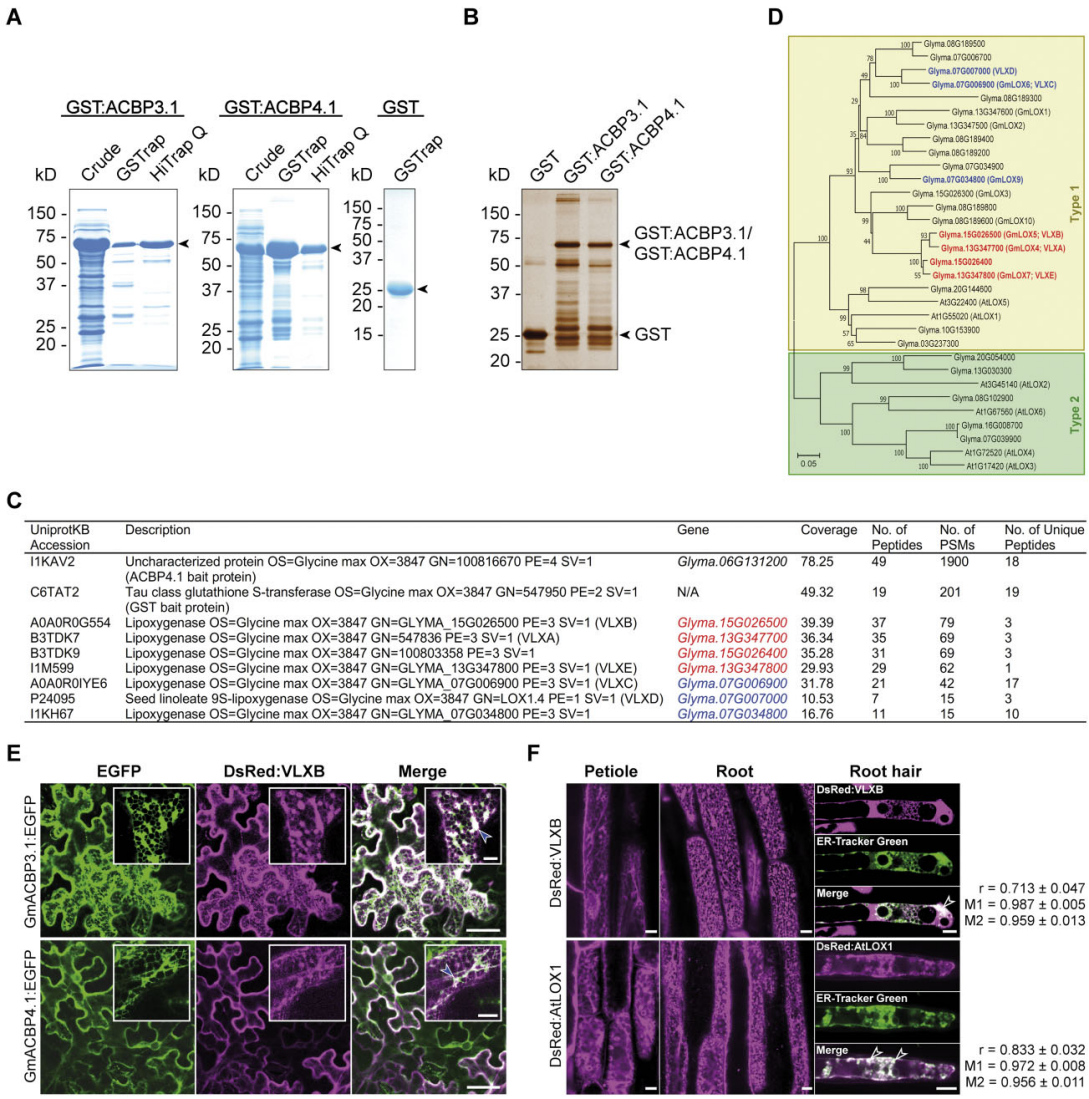
GmACBP3 and GmACBP4 interact with type-1 LOXs. A, Expression and purification of GST-fusion proteins. GST:GmACBP3.1 and GST:GmACBP4.1 were expressed in *E. coli* and purified from soluble fractions on GSTrap HP affinity and HiTrap Q HP anion-exchange chromatography columns. GST alone was purified on a GSTrap HP affinity column and served as a control in pull-down assays. Arrowheads indicate target bands of 62-kD GST:ACBP3.1 (apparent: 70 kD), 63-kD GST:ACBP4.1 (apparent: 70 kD), and 28-kD GST. B, GST pull-down assays. Purified bait proteins were immobilized to glutathione agarose for capturing potential interactors from crude soybean soluble proteins. A silver-stained SDS-PAGE gel indicates prey proteins in eluates. Arrowheads indicate target bands of 62-kD GST:ACBP3.1 (apparent: 70 kD), 63-kD GST:ACBP4.1 (apparent: 70 kD), and 28-kD GST. C, LC–MS/MS identification of prey proteins from GST:GmACBP4.1 pull-down assays. Confident proteins were identified using a target–decoy approach with a reversed database, strict false-discovery rate of <1% at peptide and PSM levels. Peptides of seven type-1 LOXs were identified. D, Neighbor-joining phylogenetic tree of soybean and Arabidopsis LOX homologs. Bootstrap values with 2,000 repetitions (%) are given at respective nodes. The scale bar indicates the distance scale (substitutions per site). Identified LOXs from LC–MS/MS as listed in (C) are shown in red and blue. See Supplemental Files S1, S2. E, GmACBP3.1:EGFP and GmACBP4.1:EGFP colocalization with DsRed:VLXB in agroinfiltrated *N. benthamiana* leaf epidermal cells. Signals were colocalized at the ER cisternae (blue arrowheads) and ER tubules. Bars = 50 or 10 μm (insets). F, Subcellular localization of DsRed:VLXB and DsRed:AtLOX1 in transgenic Arabidopsis by confocal laser scanning microscopy. In petioles and roots of 2-week-old plants, signals were detected at the tubular ER and ER cisternae. In root hair cells of 7-day-old seedlings, DsRed-fusion proteins were colocalized with the ER-Tracker signals at the membrane of ER-derived vesicles (open arrowheads). Pearson’s correlation coefficient ^®^, Manders’ overlap coefficients M1 (fraction of ER-Tracker Green overlapping DsRed), and M2 (fraction of DsRed overlapping ER-Tracker Green) were computed from regions of interest of 400 × 100 pixels. Values represent the mean ± sem of 10 cells. Bars = 10 μm.

As salt-induced alternative splicing of Class II *ACBPs* affected the ANK domain (Figure 1), this response may attenuate interaction of Class II ACBPs with unknown protein(s) as a signaling process. To identify the potential interactors, glutathione *S*-transferase (GST)-fusion proteins of ACBP3.1 and ACBP4.1 (Figure 3, A) were used as baits to capture prey proteins from soybean crude extracts (Figure 3, B). The 100% homology of the ANK domain of ACBP3 and ACBP4 implicates similarity of their protein–protein interaction network (Supplemental Figure S1). The ACBP4 interactors were identified by liquid chromatography–tandem mass spectrometry (LC–MS/MS) analysis of the eluates, which also confirmed the ACBP4 identity (Figure 3, C), given the retarded electrophoretic mobility of ACBP3 and ACBP4 (Figures 1, D, 2, C). Four of the first 10 proteins with the highest peptide spectrum matches (PSMs) belonged to LOXs, while three other LOXs were identified with lower PSMs (Figure 3, C). Phylogenetic analysis indicated that these seven LOXs were evolutionarily closer to the extraplastidial type-1 LOXs as represented by Arabidopsis AtLOX1 and AtLOX5, than the plastidial type-2 LOXs (Figure 3, D and Supplemental File S1). VLXB (Glyma.15G026500), which was detected with the most PSM (Figure 3, C), and its best Arabidopsis homolog (AtLOX1; sharing 70% protein homology) were selected for further investigations.

Confocal laser scanning microscopy indicated that GmACBP3.1:EGFP and GmACBP4.1:EGFP colocalized with DsRed:VLXB at the ER cisternae and tubular ER network of *N. benthamiana* leaf epidermal cells (Figure 3, E). While the seven GmACBP4.1-interacting type-1 LOXs were predicted to be cytosolic proteins without sorting or membrane-spanning sequences (Supplemental Figure S3, B), the ER signals of DsRed:VLXB (Figure 3, E) were attributed to its interaction with ER-localized GmACBP3.1:EGFP and GmACBP4.1:EGFP (Figure 2). DsRed:VLXB and DsRed:AtLOX1 also decorated similar ER structures of petiole and root cells in transgenic Arabidopsis (Figure 3, F). Colocalization of DsRed-fusion proteins with the ER-Tracker was indicated by the Pearson’s correlation coefficient and Manders’ overlap coefficients (Figure 3, F). Possibly, the ER-localized AtACBP1 and AtACBP2 could form an equivalent interaction interface for DsRed:VLXB and DsRed:AtLOX1, given 84% homology between the ANK domain of soybean and Arabidopsis Class II ACBPs (Supplemental Figure S1). Taken together, Class II ACBPs were shown to sequester VLXB and AtLOX1 at the ER.

### ANK and ACB domains of Class II GmACBPs facilitate VLXB interaction

**Figure 4:**
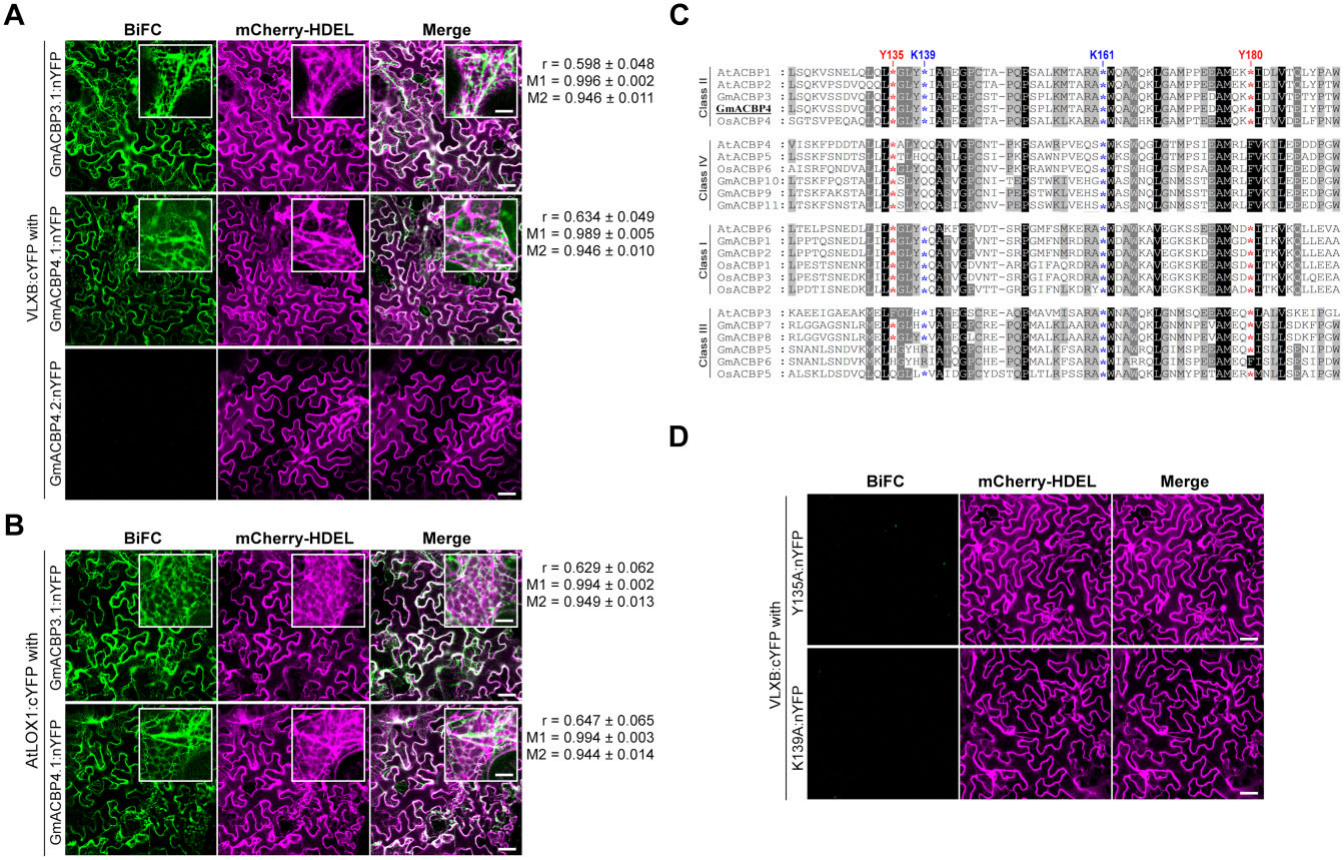
Both ANK and ACB domains of GmACBP4 are essential for VLXB interaction at the ER. *Nicotiana benthamiana* leaf epidermal cells were agroinfiltrated with split-YFP (nYFP and cYFP) fusion constructs together with the mCherry-HDEL ER marker as a transfection control. BiFC and mCherry-HDEL signals were examined by confocal laser scanning microscopy. Pearson’s correlation coefficient (*r*), Manders’ overlap coefficients M1 (fraction of YFP overlapping mCherry), and M2 (fraction of mCherry overlapping YFP) were computed from regions of interest of 150 × 150 pixels. Values represent the mean ± sem of 10 cells. Bars = 50 or 10 μm (insets). A, VLXB:cYFP was transfected with nYFP-fusion proteins of native forms (GmACBP3.1 and GmACBP4.1) and splice variant (GmACBP4.2). B, AtLOX1:cYFP was transfected with GmACBP3.1:nYFP and GmACBP4.1:nYFP. C, Amino acid alignment of the ACB domain of *A. thaliana*, *G. max*, and *Oryza sativa* ACBPs featuring four conserved residues (Tyr-135, Lys-139, Lys-161, and Tyr-180) selected for site-directed mutagenesis (as indicated by asterisks). Sequences were aligned using Clustal W version 1.83. Residues are blocked on a black (100% conserved), dark gray (>75% conserved), and light gray (50%–74% conserved) background. D, VLXB:cYFP was transfected with Y135A and K139A mutants of GmACBP4.1:nYFP.

To define the critical elements of Class II GmACBPs for VLXB interaction, fusion proteins of ACBPs with the N-terminal half of split yellow fluorescent protein (nYFP) and VLXB with the C-terminal half of split-YFP (cYFP) were coexpressed in *N. benthamiana* leaves for bimolecular fluorescence complementation (BiFC) assays. Confocal laser scanning microscopy revealed that VLXB:cYFP and nYFP-fusion proteins of the native forms (GmACBP3.1 and GmACBP4.1) reconstituted the fluorescence signals, which colocalized with the mCherry-HDEL ER marker (Figure 4, A). Similar signals were observed when VLXB:cYFP was replaced with AtLOX1:cYFP (Figure 4, B), suggesting conserved interaction of Class II ACBPs with type-1 LOXs in soybean and Arabidopsis. In contrast, nYFP-fused splice variants did not complement with VLXB:cYFP, while mCherry-HDEL as an internal reference indicated successful transfection (Figure 4, A and Supplemental Figure S4, A and B).

To investigate whether a functional ACB domain of GmACBP4.1 is required for VLXB interaction, the GmACBP4.1 sequence was modified by single Ala substitution at each of the four selected residues, Tyr-135, Lys-139, Lys-161, and Tyr-180, which were conserved among Arabidopsis, soybean and rice Class II ACBPs (Figure 4, C). Ala substitution at the equivalent sites had previously abolished C16:0-CoA binding of recombinant AtACBP2 (Chye et al., 2000). BiFC assays indicated that none of these GmACBP4.1 mutants interacted with VLXB (Figure 4, D and Supplemental Figure S4, B). Immunoblot analysis verified the expression of VLXB:cYFP and nYFP-fusion proteins in *N. benthamiana* leaves (Supplemental Figure S4, C), suggesting that the absence of YFP reconstitution by the splice variants and protein mutants reflected the lack of VLXB interaction (Figure 4, A and D and Supplemental Figure S4, A and B).

**Figure 5:**
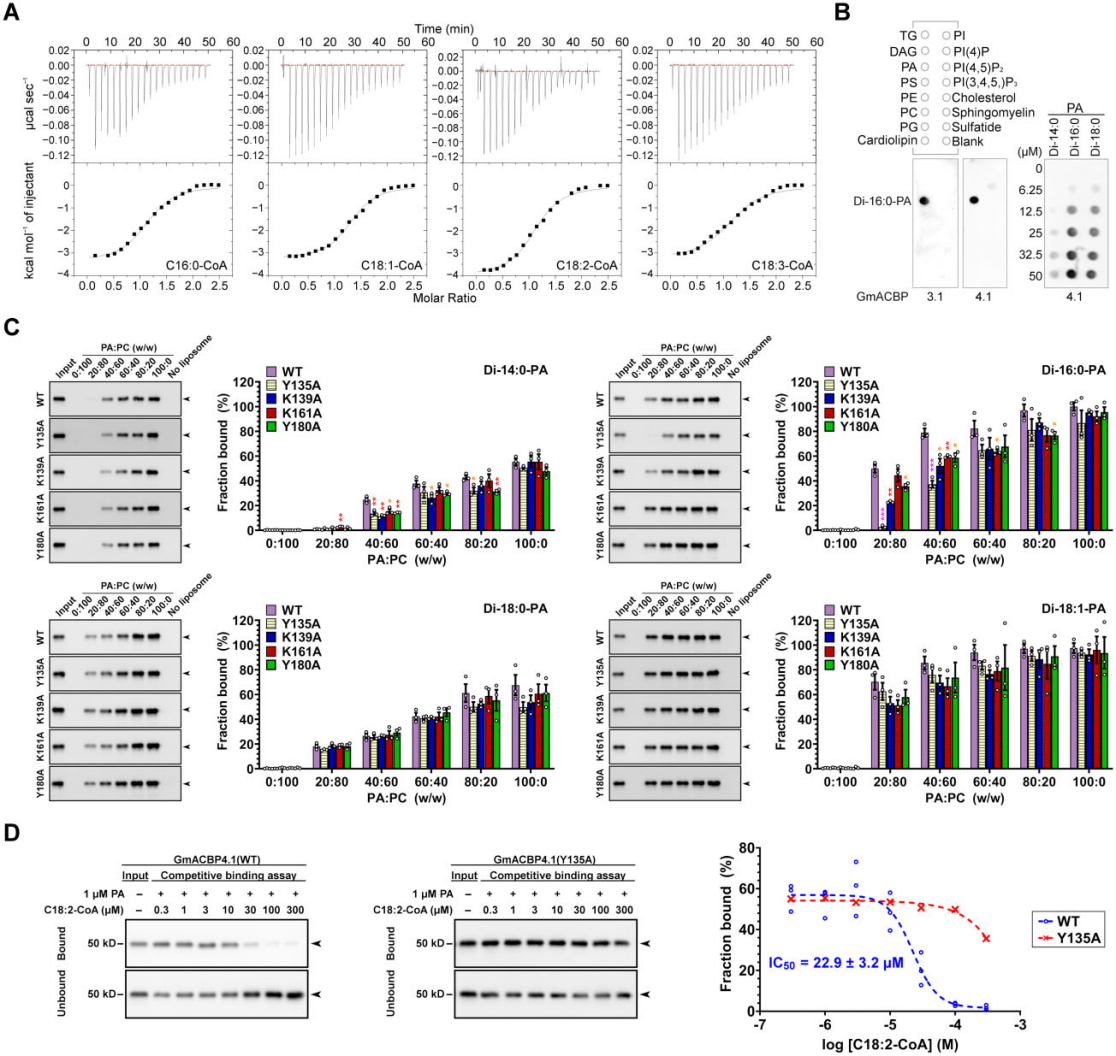
Long-chain acyl-CoAs and phosphatidic acid compete for the ligand-binding site of GmACBP4. A, ITC analysis for acyl-CoA binding by titrating 250 μL of 15 μM (His)_6_-GmACBP4.1_33–354_ with 20 injections of 1.8-μL aliquots of 200 μM C16:0-, C18:1-, C18:2-, or C18:3-CoAs at 25°C. Representative binding isotherms including raw heating power over time (upper panels) and integrated area of each injection after background correction (lower panels) are shown. Similar results were obtained from three independent experiments. Binding constants and thermodynamic parameters are given in Supplemental Table S1. B, Protein–lipid overlay assays. Commercial filter strips with prespotted lipids (left panels) and nitrocellulose filter strips spotted with various concentrations of PA species (right panel) were incubated with 0.5 μg mL^−1^ (His)_6_-GmACBP3.1_33–346_ or (His)_6_-GmACBP4.1_33–354_. Protein binding was detected by immunoblotting using anti-ACBP3.1 antibodies. DAG, diacylglycerol; PE, phosphatidylethanolamine; PG, phosphatidylglycerol; PI, phosphatidylinositol; PS, phosphatidylserine; TG, triglyceride. C, Liposome association assays for PA binding. Liposomes (100 μg) constituting PA (di-14:0, di-16:0, di-18:0, or di-18:1) and PC (di-16:0-PC) mixtures at 0–100% (w/w) ratios were incubated with 0.5 μg of (His)_6_-GmACBP4.1_33–354_ including the WT and mutants (Y135A, K139A, K161A, and Y180A). Associated proteins were detected by immunoblotting using anti-ACBP3 antibodies. Representative blots are shown after similar results were obtained from three independent experiments. Input lanes represent 50% protein. Arrowheads indicate 40-kD target bands (apparent: 50 kD). The liposome-associated protein fraction was quantified densitometrically. Each bar represents the mean ± sem from three independent experiments. Asterisks indicate statistically significant (*P < 0.05; **P < 0.01; ***P < 0.001) difference from WT at the same PA:PC ratio by Student’s *t* test (Supplemental Data Set S1). D, Protein–lipid bead binding assays with competing acyl-CoAs. Agarose beads coated with 1 μM PA were incubated with 2.5 μg mL^−1^ (His)_6_-GmACBP4.1_33–354_ (WT or Y135A mutant) in the presence of competing C18:2-CoA at various concentrations. Bound and unbound WT (left panel) and Y135A (middle panel) were detected by immunoblotting using anti-GsACBP3 antibodies. Input lanes represent 50% protein. Arrowheads indicate target bands. The PA-bound protein fraction was quantified densitometrically. The half maximal inhibitory concentration (IC_50_) of the WT is the mean ± sem from three independent experiments.

To confirm that the wild-type (WT) but not the four mutants of GmACBP4.1 binds with acyl-CoAs, recombinant proteins without the TM domain (GmACBP4.1_33–354_) were produced (Supplemental Figure S5, A) for in vitro binding assays. First, binding of the WT with long-chain (C16:0, C18:1, C18:2, and C18:3) acyl-CoAs was quantified by isothermal titration calorimetry (ITC; Figure 5, A). The binding isotherms showed that injection of acyl-CoAs into protein solution was exothermic in nature and the magnitude of heat release was similar among the four species tested (Figure 5, A and Supplemental Table S1). The dissociation constant (*K*_D_) for WT interaction with the four acyl-CoAs indicated their comparable affinities within the micromolar range (Supplemental Table S1). The stoichiometry (*n*) values approximated to 1, suggesting interaction of one protein with single acyl-CoA molecule (Supplemental Table S1). ITC binding isotherms revealed that association of all mutants with the four acyl-CoA species was disrupted because a typical sigmoidal curve was not obtained from the four mutants for the calculation of binding constants and thermodynamic kinetics (Supplemental Figure S6). Hence, the ANK domain and ACB activity of GmACBP4 were shown to be crucial for VLXB interaction. The same criteria for GmACBP3–VLXB interaction were deduced, given that the ANK and ACB domains of Class II GmACBPs are 100% and 99% homologous, respectively (Supplemental Figure S1).

### PA competes with acyl-CoAs for binding with GmACBP4

Besides acyl-CoAs, the affinity of GmACBP3.1_33–346_ and GmACBP4.1_33–354_ to membrane lipids was assessed by protein–lipid overlay assays using commercial filter strips spotted with 15 neutral and polar lipids, among which di-16:0-PA was found to be the sole ligand (Figure 5, B, left panel). Two more PA species (di-14:0 and di-18:0) were shown to bind with GmACBP4.1_33–354_, albeit at lower affinity (Figure 5, B, right panel). Subsequently, PA binding of the WT and four mutants of GmACBP4.1_33–354_ was compared by liposome association assays (Figure 5, C). As the WT did not interact with di-16:0-phosphatidylcholine (PC) on filter strips (Figure 5, B, left panel), it was mixed with PA at different w/w ratios to constitute liposomes representing a progressive rise in PA from 0% to 100% (Figure 5, C). Similar to the observation on filter strips (Figure 5, B, right panel), liposome association assays revealed a descending order of affinity to the WT as follows: di-16:0-PA ≈ di-18:1-PA > di-18:0-PA > di-14:0-PA (Figure 5, C). The single amino acid substitution in the four mutants subtly weakened PA binding, except for a greater decline in Y135A association with 20% (w/w) di-16:0-PA liposomes (Figure 5, C).

Although Y135A, K139A, K161A, and Y180A substitutions of GmACBP4.1_33–354_ were more impactful on binding with acyl-CoAs (Supplemental Figure S6) than PA (Figure 5, C), all lipids might interact with GmACBP4.1_33–354_ via its ACB domain as the only-identified lipid-binding site (Figure 1, A). To validate this assumption, the binding of the WT and Y135A mutant to PA beads was investigated in the presence of C18:2-CoA (Figure 5, D). In competitive binding assays, the fraction of PA-bound WT diminished with more C18:2-CoA, of which the half-maximal inhibitory concentration (IC_50_) was 22.9 μM. Unlike the WT, Y135A binding to PA beads was less affected by C18:2-CoA (Figure 5, D). Collectively, we found that PA and acyl-CoAs are competitive ligands for GmACBP4. As the four GmACBP4 mutants bound with PA (Figure 5, C) but barely with acyl-CoAs (Supplemental Figure S6), the lack of VLXB interaction with these four mutants in BiFC assays (Figure 4, D and Supplemental Figure S4) was attributed to their weaker acyl-CoA affinity.

### Specific acyl-CoA species facilitate GmACBP4 interaction with protein partners

**Figure 6:**
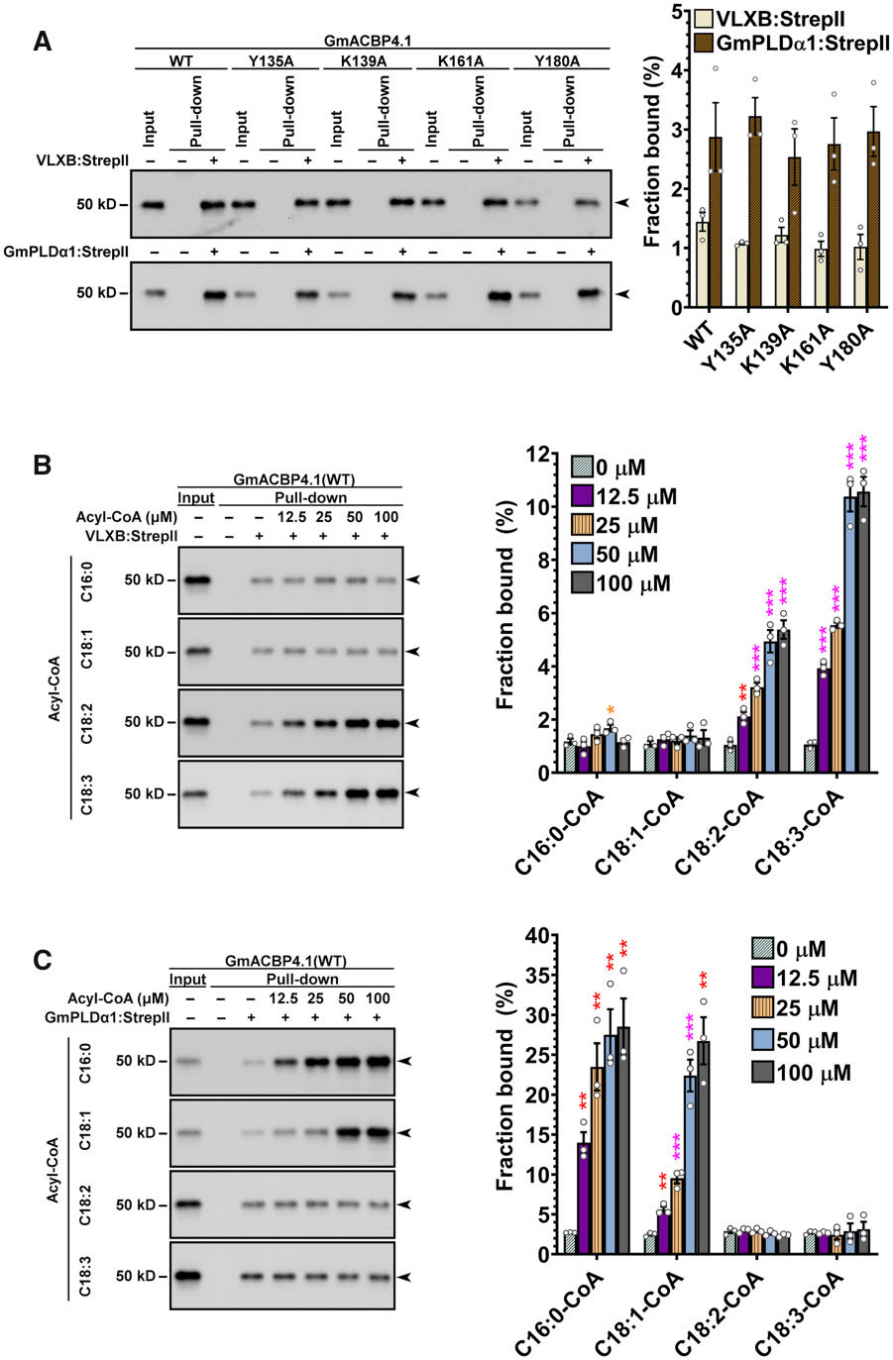
Specific acyl-CoA species facilitate GmACBP4 interaction with VLXB and GmPLDα1. A, Strep-Tactin pull-down assays. Strep-Tactin beads with/without prebound VLXB:StrepII and GmPLDα1:StrepII proteins were incubated with equimolar concentration of (His)_6_-GmACBP4.1_33–354_ including the WT and mutants (Y135A, K139A, K161A, and Y180A). (His)_6_-GmACBP4.1_33–354_ were detected in eluates by immunoblotting using anti-ACBP3 antibodies. Representative blots are shown after similar results were obtained from three independent experiments. Input lanes represent 1% protein. Arrowheads indicate target bands of 40-kD (His)_6_-GmACBP4.1_33–354_ (apparent: 50 kD). The bound protein fraction was quantified densitometrically. Each bar represents the mean ± sem from three independent experiments. No significant (*P* > 0.05) difference between the WT and mutants was obtained by Student’s *t* test (Supplemental Data Set S1). B–C, Strep-Tactin pull-down assays with supplemented acyl-CoAs. Strep-Tactin beads with/without prebound VLXB:StrepII in (B) and GmPLDα1:StrepII in (C) were incubated with (His)_6_-GmACBP4.1_33–354_ (WT) in the presence of serial dilutions of C16:0-, C18:1-, C18:2-, or C18:3-CoA. Input lanes represent 5% proteins in (B) or 10% proteins in (C). Arrowheads indicate target bands of 40-kD (His)_6_-GmACBP4.1_33–354_ (apparent: 50kD). Each bar represents the mean ± sem from three independent experiments. Asterisks indicate statistically significant (*P < 0.05; **P < 0.01; ***P < 0.001) difference from the control without acyl-CoA by Student’s *t* test (Supplemental Data Set S1).

To pinpoint the acyl-CoA species that facilitated the GmACBP4–VLXB interaction, VLXB:StrepII was expressed and purified for pull-down assays (Supplemental Figure S5, B). Based on our previous report of interaction between Class II AtACBP1 and PLDα1 in Arabidopsis (Du et al., 2013a), GmPLDα1:StrepII was also prepared to verify its interaction with GmACBP4.1_33–354_ (Supplemental Figure S5, B). Strep-Tactin pull-down assays revealed that GmACBP4.1_33–354_ was captured by immobilized VLXB:StrepII and GmPLDα1:StrepII (Figure 6, A). Without acyl-CoAs, the StrepII-tagged proteins captured similar amount of the WT and four mutants of GmACBP4.1_33–354_ (Figure 6, A). C18:2-CoA increased WT binding with VLXB:StrepII by up to four-fold in a concentration-dependent manner (Figure 6, B). A nine-fold increase was achieved with 100 µM C18:3-CoA, while the effect of C16:0/C18:1-CoA supplementation was negligible (Figure 6, B). The four acyl-CoAs did not facilitate VLXB:StrepII interaction with Y135A, K139A, K161A, and Y180A mutants (Supplemental Figure S7), in agreement with their compromised acyl-CoA affinity (Supplemental Figure S6). Conversely, C16:0/C18:1-CoAs facilitated WT binding with GmPLDα1:StrepII by up to nine-fold, while C18:2/C18:3-CoAs did not influence WT interaction with GmPLDα1 (Figure 6, C). Taken together, specific acyl-CoAs were shown to facilitate GmACBP4 interaction with VLXB and GmPLDα1.

### Overexpression of native and variant forms leads to reciprocal salinity phenotypes

**Figure 7:**
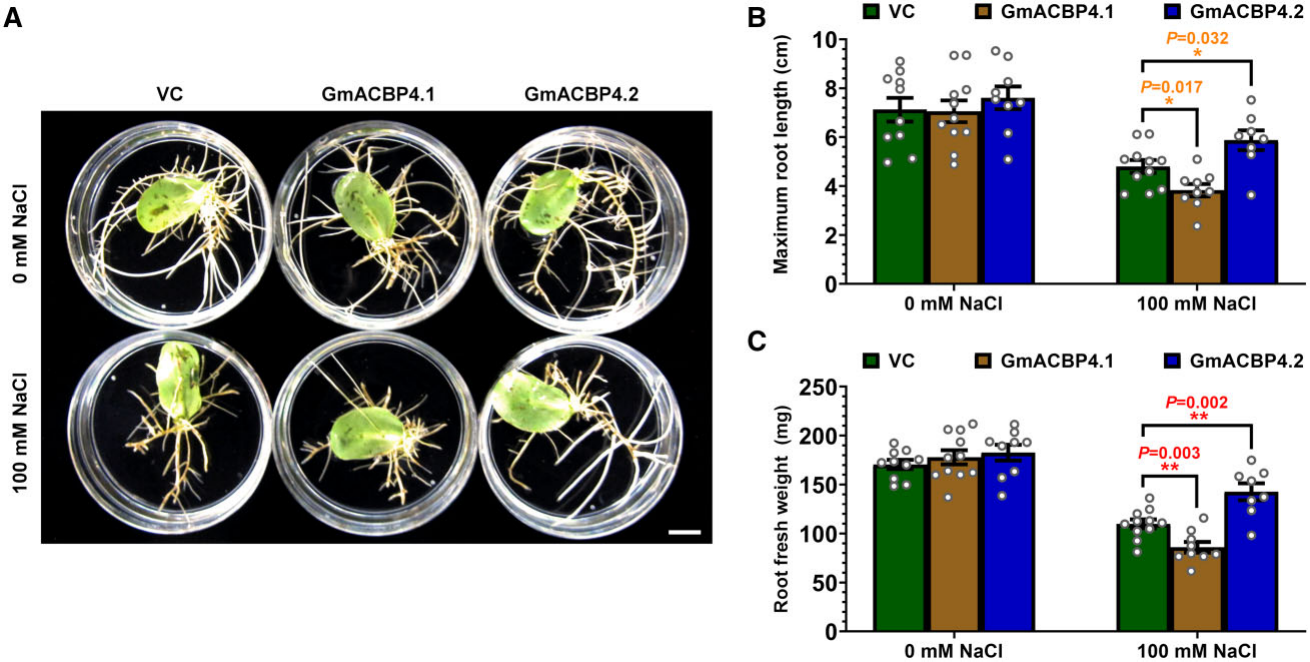
Overexpression of *GmACBP4.1* and *GmACBP4.2* in soybean hairy roots produces reciprocal phenotypes under high salinity. Transgenic C08 soybean hairy roots were generated by *A. rhizogenes*-mediated transformation of cotyledons with plasmids harboring *35Spro:GmACBP4.1*, *35Spro:GmACBP4.2*, and VC. A, Representative photo of hairy roots grown with/without 100 mM NaCl for 10 days. Bar = 1 cm. B–C, Salt sensitivity was assessed by measuring maximum root length as shown in (B) and root fresh weight in (C) after 10-day treatment. Each bar represents the mean of 8–11 PCR-positive (Supplemental Figure S9) hairy root samples ± sem. Asterisks indicate statistically significant difference (*P < 0.05; **P < 0.01) from VC by Student’s *t* test (Supplemental Data Set S1).

The physiological relevance of salt-induced alternative splicing of Class II *GmACBPs* was addressed in planta by transforming C08 soybean hairy roots with *35Spro:GmACBP4.1* and *35Spro:GmACBP4.2* for overexpression of the native and variant forms, respectively. Positive transformants were verified by PCR (Supplemental Figure S8). Under normal conditions, *GmACBP4.1*- and *GmACBP4.2*-transformed roots were indistinguishable from the vector control (VC; Figure 7, A). Salinity severely suppressed growth of *GmACBP4.1*-transformed roots (Figure 7, A), of which the average maximum length (Figure 7, B) and fresh weight (Figure 7, C) were lower than the VC. In contrast, *GmACBP4.2*-transformed roots were more tolerant to NaCl treatment (Figure 7, A), exhibiting increase in maximum length (Figure 7, B) and fresh weight (Figure 7, C) in comparison with the VC.

**Figure 8:**
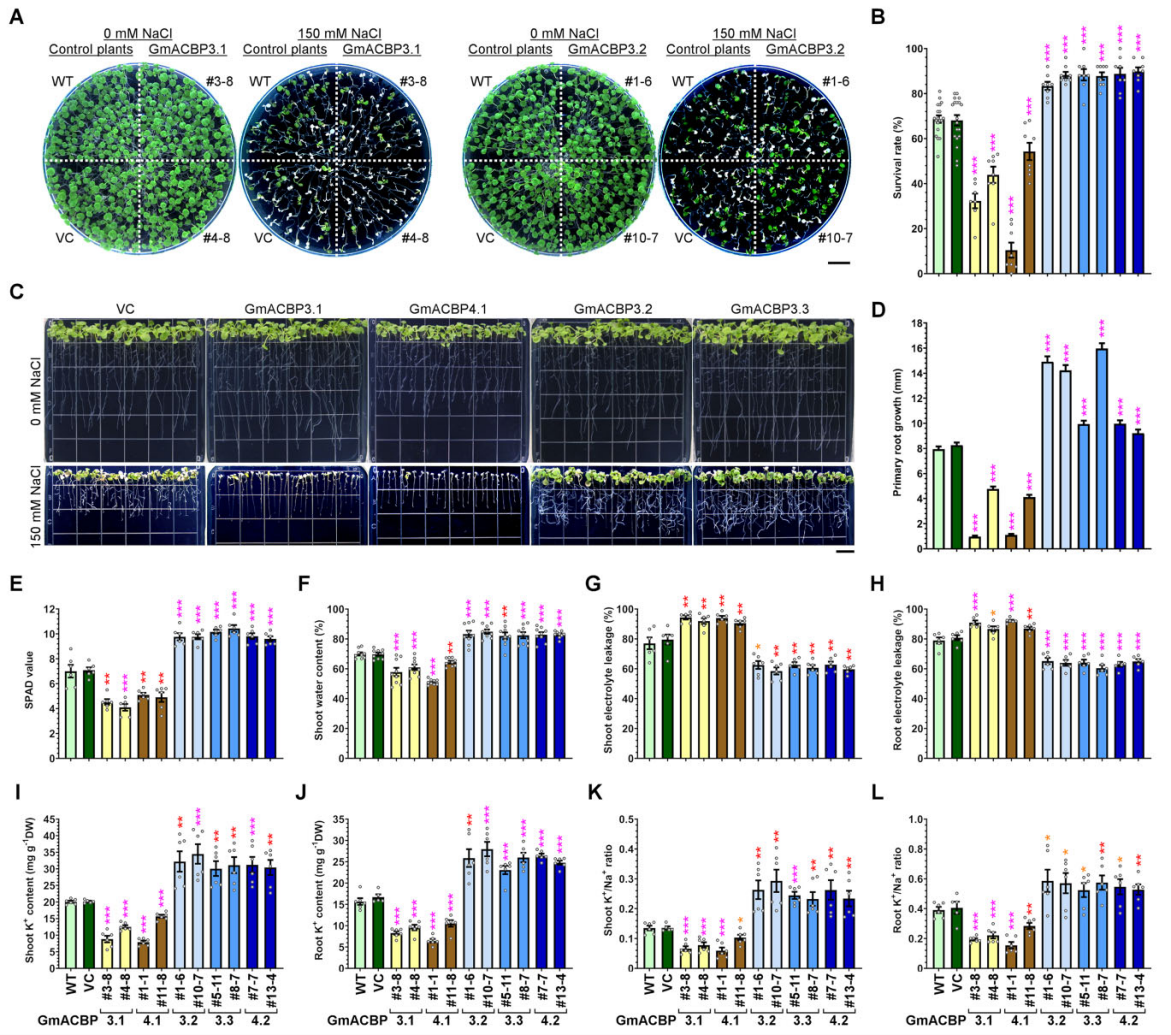
Transgenic Arabidopsis expressing native forms and splice variants of Class II GmACBPs exhibit reciprocal phenotypes under high salinity. High-salinity phenotypes of transgenic Arabidopsis expressing the native forms (GmACBP3.1 and GmACBP4.1) and splice variants (GmACBP3.2, GmACBP3.3, and GmACBP4.2), the WT and VC were examined. Asterisks indicate statistically significant (*P < 0.05; **P < 0.01; ***P < 0.001) difference from WT under the same treatment by Student’s *t* test (Supplemental Data Set S1). A–D, Phenotypes of Arabidopsis seedlings. Four-day-old seedlings were transferred to MS plates with/without 150 mM NaCl. Representative photos in (A) and (C) were taken after 7 and 8 days, respectively. Bar = 1 cm. Survival rate after 14-day treatment in (B) is the mean ± sem calculated from at least seven test plates. Primary root growth in (D) after 8-day treatment is the mean ± sem of 75 seedlings. E–L, Phenotypes of hydroponically-grown Arabidopsis. Four-week-old plants were treated in fresh hydroponic solution with 150 mM NaCl for 10 days. Chlorophyll content measured by the SPAD meter in (E), shoot and root electrolyte leakage in (G–H), K+ content in (I–J), and K+/Na+ ratio in (K–L) are the mean ± sem of six plants. Shoot water content in (F) is the mean ± sem of nine plants.

As Class II GmACBPs also interacted with AtLOX1 (Figure 4, B), we examined if the reciprocal salinity response of *GmACBP4.1*- and *GmACBP4.2*-transformed soybean hairy roots could be observed similarly in transgenic Arabidopsis. To this end, the native and variant forms of GmACBP3 and GmACBP4 were expressed under the control of *35S* promoter in Arabidopsis for salt tolerance tests. In comparison with the WT and VC, transgenic seedlings expressing the native and variant forms were more sensitive and tolerant, respectively, whereas all lines grew normally under nonstressed conditions (Figure 8, A and Supplemental Figure S9, A). A lower percentage of seedlings expressing GmACBP3.1 and GmACBP4.1 survived under salt stress, while GmACBP3.2, GmACBP3.3, and GmACBP4.2 expression improved the survival rate (Figure 8, B). Contrary to the stunted root development of salt-stressed seedlings expressing the native forms, a more profuse root system was seen upon overexpression of the splice variants under salinity (Figure 8, C). Primary root growth of GmACBP3.1- and GmACBP4.1-transformed seedlings were slower than the WT and VC under salt stress, while primary root elongation of seedlings expressing the splice variants was less affected by NaCl treatment (Figure 8, D).

In addition, when 4-week-old Arabidopsis plants were treated in hydroponics with 150 mM NaCl, transgenic plants expressing GmACBP3.1 and GmACBP4.1 resembled younger seedlings of the same genotype in their salt hypersensitivity, as indicated by greater loss of leaf chlorophyll (Figure 8, E) and shoot water (Figure 8, F) content than the WT and VC. Consistently, these transgenic plants exhibited higher electrolyte leakage in shoots (Figure 8, G) and roots (Figure 8, H), which also showed lower K^+^ content (Figure 8, I and J) and K^+^/Na^+^ ratio (Figure 8, K and L) under salinity. In contrast, NaCl treatment led to milder phenotypes in transgenic Arabidopsis expressing the splice variants, which had higher leaf chlorophyll (Figure 8, E) and shoot water (Figure 8, F) content than the WT and VC, concomitant with lower shoot (Figure 8, G) and root (Figure 8, H) electrolyte leakage. Arabidopsis plants expressing the splice variants also contained higher K^+^ content (Figures 8, I and J) and K^+^/Na^+^ ratio (Figure 8, K and L) in shoots and roots than the WT and VC under salinity. Under nonstressed conditions, all transgenic Arabidopsis did not differ from the WT in chlorophyll and shoot water content, electrolyte leakage, K^+^ content, and K^+^/Na^+^ ratio (Supplemental Figure S9, B–G). Taken together, overexpression of native Class II GmACBPs caused salt hypersensitivity of soybean hairy roots and Arabidopsis, while transgenic plants expressing the splice variants exhibited higher salt tolerance. Hence, salt-induced alternative splicing of *GmACBP3* and *GmACBP4* is an adaptive response to aid plant survival under adversity.

### Class II GmACBPs regulate FA composition, lipid peroxidation, and LOX activity

**Figure 9:**
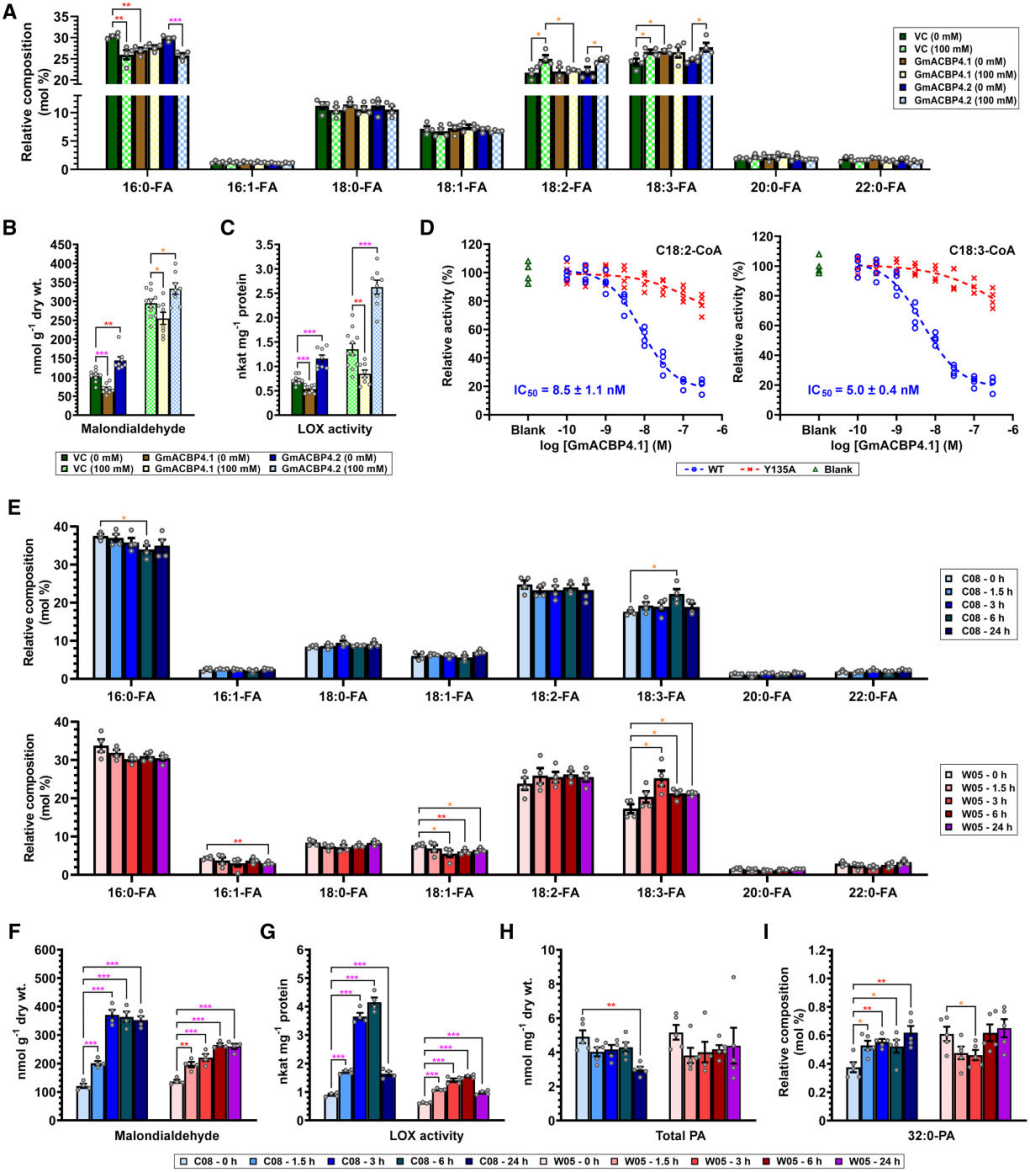
Class II GmACBPs modulate LOX activity in soybean roots during salinity response. A–C, Transgenic soybean hairy roots were generated by *A. rhizogenes*-mediated transformation of cotyledons with plasmids harboring *35Spro:GmACBP4.1*, *35Spro:GmACBP4.2*, and VC. Hairy roots were grown with/without 100 mM NaCl for 10 days. FA composition in (A) is the mean of four hairy root samples ± sem. MDA content in (B) is the mean of eight to 11 hairy root samples ± sem. LOX activity in (C) is the mean of 8–11 hairy root samples ± sem. Asterisks indicate statistically significant (*P < 0.05; **P < 0.01; ***P < 0.001) difference by Student’s *t* test (Supplemental Data Set S1). D, In vitro enzyme assays for the inhibitory effect of GmACBP4.1 on VLXB. VLXB:StrepII was incubated with the WT or Y135A mutant of (His)_6_-GmACBP4.1_33–354_ in the presence of C18:2- or C18:3-CoA, prior to LOX activity assays. The activity was normalized to the blank without GmACBP4.1. The half maximal inhibitory concentration (IC_50_) of the WT is the mean ± sem from four independent experiments. E–G, Three-week-old wild W05 and cultivated C08 soybean plants were treated in hydroponic solution with 0.9% (w/v) NaCl. Roots were harvested at 0, 1.5, 3, 6, and 24 h for subsequent analyses of FA composition in (E), MDA content in (F), and LOX activity in (G). Each bar represents the mean of four plants ± sem. Asterisks indicate statistically significant (*P < 0.05; **P < 0.01; ***P < 0.001) difference from 0 h by Student’s *t* test (Supplemental Data Set S1). H–I, PA analysis. Total PA content in (H) and 32:0-PA level (mol % of total PA) in (I) were analyzed after treatment of cultivated C08 and wild W05 soybean roots in hydroponic solution with 0.9% (w/v) NaCl for 0, 1.5, 3, 6, and 24 h. Each bar represents the mean of five plants ± sem. Asterisks indicate statistically significant (*P < 0.05; **P < 0.01) difference from 0 h by Student’s *t* test (Supplemental Data Set S1).

To correlate the different salinity phenotypes with potential alterations in lipid metabolism, FAs in soybean hairy roots were first analyzed by gas chromatography–mass spectrometry (GC–MS). The FA composition of *GmACBP4.2*-transformed roots did not differ from that of the VC with/without NaCl treatment (Figure 9, A). Under nonstressed conditions, *GmACBP4.1*-transformed roots produced more C18:3-FA but less C16:0-FA than the VC (Figure 9, A). Salt stress upregulated C18:2-FA and C18:3-FA and lowered C16:0-FA in *GmACBP4.2*-transformed roots and the VC (Figure 9, A). In contrast, NaCl treatment did not change FA composition of *GmACBP4.1*-transformed roots, which thus had lower C18:2-FA than *GmACBP4.2*-transformed roots and the VC under salinity (Figure 9, A). In Arabidopsis, overexpression of Class II GmACBPs did not alter long-chain (C16 and C18) FAs in roots (Supplemental Figure S10, A) and shoots (Supplemental Figure S10, B). Higher levels of very-long-chain (C20:0 and C22:0) FAs, however, were observed in roots of most transgenic lines expressing the native and variant forms of Class II GmACBPs (Supplemental Figure S10, A).

To investigate how Class II GmACBPs modulate LOX activity in planta, Class II GmACBP-overexpressing roots were analyzed by thiobarbituric acid-reactive substance assays for their content of malondialdehyde (MDA), which indicated the formation of polyunsaturated FA (PUFA) hydroperoxides (Heath and Packer, 1968). Under normal conditions, *GmACBP4.1*- and *GmACBP4.2*-transformed roots exhibited 33% lower and 39% higher MDA than the VC, respectively (Figure 9, B). This difference diminished to −14% in *GmACBP4.1*-transformed roots and +13% in *GmACBP4.2*-transformed roots under salinity (Figure 9, B). The homogenates of transgenic roots were further assayed for in vitro LOX activity using C18:2-FA as the substrate (Figure 9, C). Consistently, *GmACBP4.1*- and *GmACBP4.2*-transformed roots exhibited 26% lower and 61% higher LOX activity than the VC under normal conditions, respectively (Figure 9, C). The difference became more evident under salinity, that is, −37% in *GmACBP4.1*-transformed roots and +94% in *GmACBP4.2*-transformed roots (Figure 9, C). To confirm the inhibition of VLXB activity by GmACBP4.1 interaction, recombinant VLXB:StrepII and GmACBP4.1_33–354_ (WT and Y135A mutant) were used for in vitro enzyme assays (Figure 9, D). The VLXB activity declined in the presence of more WT (Figure 9, D). The IC_50_ was 8.5 and 5.0 nM with 10 µM C18:2-CoA and C18:3-CoA, respectively (Figure 9, D), both of which could facilitate GmACBP4–VLXB interaction (Figure 6, B). Comparatively, the inhibitory effect of Y135A was attenuated (Figure 9, D), owing to its compromised acyl-CoA binding (Supplemental Figure S6) affecting VLXB interaction (Supplemental Figure S7).

While *GmACBP4.2* overexpression boosted lipid peroxidation (Figure 9, B) and LOX activity (Figure 9, C) in soybean hairy roots, C08 and W05 roots were also compared as the former elicited more pronounced alternative splicing of Class II *ACBPs* in the salinity response (Figure 1, B–D). Time-resolved analyses of lipid metabolism in the two contrasting genotypes will better establish the transient role of variant proteins, which dominated at 3 and 6 hpt (Figure 1, D). First, FA composition analysis showed higher C18:3-FA and lower C16:0-FA in C08 roots at 6 hpt (Figure 9, E, top panel). In W05 roots, C18:3-FA was upregulated at the expense of C18:1-FA at 3 hpt, and this change diminished after prolonged treatment (Figure 9, E, bottom panel). The similarity of C18:3-FA increase in both genotypes implies its irrelevance to alternative splicing (Figure 9, E), consistent with the indistinguishable FA composition of *GmACBP4.2*-transformed soybean hairy roots versus the VC (Figure 9, A). On the other hand, MDA measurement revealed that C08 roots exhibited a faster, greater response in lipid peroxidation than W05 roots (Figure 9, F). Consistently, the accumulation of splice variants coincided with the highest LOX activity in C08 roots at 3 and 6 hpt (3.7 and 4.2 nkat mg^−1^ protein), whereas the LOX activity in W05 roots (up to 1.5 nkat mg^−1^ protein) was subtly upregulated (Figure 9, G).

As the ligand-binding status of GmACBP4 governs its interaction with VLXB (Figure 6, B), PA as another ligand of Class II GmACBPs (Figure 5, B–D) was analyzed in NaCl-treated C08 and W05 roots. At 1.5, 3, and 6 hpt, the total PA content of both genotypes appeared unchanged while 32:0-PA was enriched by 39%–48% versus 0 hpt at the expense of 32:1-PA (3 hpt), 36:1-PA (6 hpt) and 40:1-PA (1.5 and 6 hpt) in C08 but not W05 (Figure 9, H and I and Supplemental Figure S11). The 24-h treatment seemed more impactful in C08 which produced 39% less PA with compositional changes in eight species, while only four were altered in W05 (Figure 9, H and I and Supplemental Figure S11).

**Figure 10:**
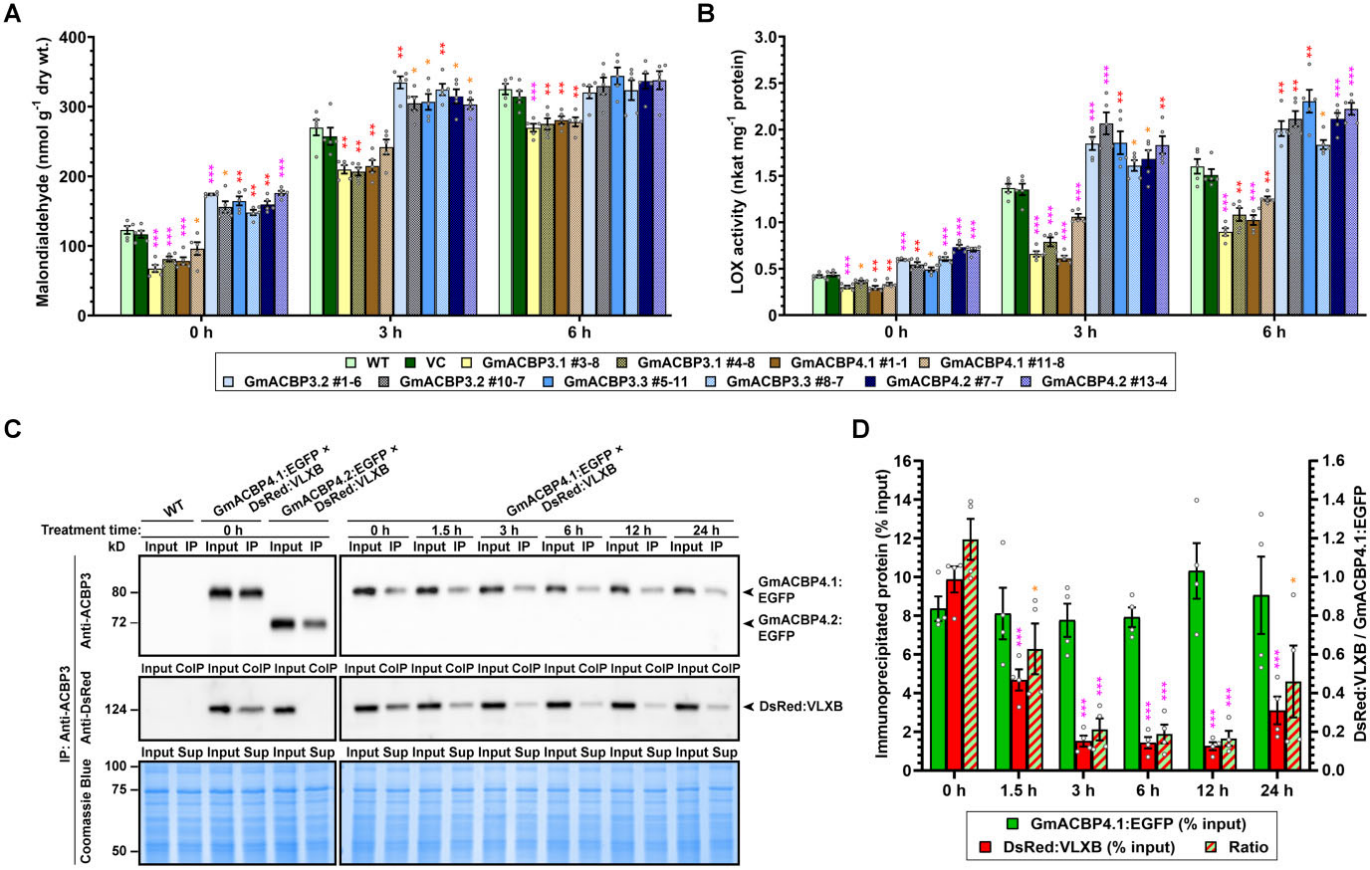
Class II GmACBPs modulate LOX activity in Arabidopsis roots during salinity response. A–B, Four-week-old Arabidopsis expressing Class II GmACBPs, the WT and VC were treated in hydroponic solution with 150 mM NaCl. Roots were harvested at 0, 3, and 6 h for subsequent analyses of MDA content in (A) and LOX activity in (B). Each bar represents the mean of five plants ± sem. Asterisks indicate statistically significant (*P < 0.05; **P < 0.01; ***P < 0.001) difference from WT at the same time point by Student’s *t* test (Supplemental Data Set S1). C, CoIP of DsRed:VLXB with GmACBP4.1:EGFP in transgenic Arabidopsis roots. Total protein extracts were immunoprecipitated with anti-ACBP3 antibodies. Immunoprecipitates and 20% input proteins were resolved on 10% SDS-PAGE and analyzed by immunoblotting using anti-ACBP3 and anti-DsRed antibodies. Left panels are representative blots of CoIP assays without NaCl treatment after similar results were obtained from two independent experiments. Arabidopsis coexpressing GmACBP4.2:EGFP and DsRed:VLXB and the WT served as negative controls. Right panels are representative blots of CoIP assays after 150 mM NaCl treatment of Arabidopsis coexpressing GmACBP4.1:EGFP and DsRed:VLXB. Similar results were obtained from four independent assays. Coomassie blue staining showed similar concentration of input and unbound proteins in the supernatant (Sup). Arrowheads indicate target bands of 66-kD GmACBP4.1:EGFP (apparent: 80 kD), 62-kD GmACBP4.2:EGFP (apparent: 73 kD) and 124-kD DsRed:VLXB. D, Densitometric analysis of immunoprecipitated GmACBP4.1:EGFP and coimmunoprecipitated DsRed:VLXB. Each bar represents the mean ± sem from four independent assays with different plants. Asterisks indicate statistically significant (*P < 0.05; **P < 0.01; ***P < 0.001) difference from 0 h by Student’s *t* test (Supplemental Data Set 1).

While the salinity phenotypes of transgenic soybean hairy roots (Figure 7) were attributed to the modulation of lipid peroxidation (Figure 9, B) and LOX activity (Figure 9, C), such correlation was next sought in Arabidopsis roots. Consistently, overexpressors of the native and variant forms contained 22%–45% lower and 20%–43% higher MDA than the WT under normal conditions, respectively (Figure 10, A). After 3-h NaCl treatment, such difference declined in overexpressors of the native (10%–23% decrease) and variant (13%–24% increase) forms (Figure 10, A). At 6 hpt, subtle (14%–17%) reduction was recorded in overexpressors of the native forms, while overexpressors of the variants exhibited the WT level of MDA (Figure 10, A). In comparison with the WT, LOX activity of Arabidopsis roots overexpressing native Class II GmACBPs was also reduced by 15%–30% at 0 hpt, 22%–55% at 3 hpt, and 22%–44% at 6 hpt (Figure 10, B). In contrast, Arabidopsis roots overexpressing the variants upregulated LOX activity, that is, 18%–75% at 0 hpt, 18%–51% at 3 hpt, and 15%–44% at 6 hpt (Figure 10, B). Thus, the native and variant forms of Class II GmACBPs were demonstrated to inhibit and stimulate LOX-catalyzed lipid peroxidation, respectively.

To explore the salt-induced effect on GmACBP4–VLXB interaction, Arabidopsis lines were crossed to coexpress DsRed:VLXB with GmACBP4.1:EGFP or GmACBP4.2:EGFP. Coimmunoprecipitation (CoIP) showed that GmACBP4.2:EGFP did not interact with DsRed:VLXB in Arabidopsis roots (Figure 10, C), similar to the results from BiFC assays (Figure 4, A). While GmACBP4.1:EGFP interacted with DsRed:VLXB in untreated roots, salinity suppressed this interaction at 1.5 hpt, and more substantially >3 hpt (Figure 10, C and D). Together, we conclude that PA signaling and alternative splicing suppress ligand-dependent interaction of Class II ACBPs with VLXB, thereby triggering lipid peroxidation during salt stress.

## Discussion

### Splice variants of Class II GmACBPs activate LOX catalysis

Despite comprehensive research on plant ACBPs, their regulation by alternative splicing has never been reported. In NaCl-treated soybean roots, three splice variants of Class II *ACBPs* arose from intron retention (Figure 1, A and B), the commonest alternative splicing event in plants (Laloum et al., 2018). C08 (Lam et al., 2010) and W05 genomic sequences (Qi et al., 2014) exhibit no polymorphism within the splicing regions. The more abundant splice variants in C08 than W05 roots may have arisen from their difference in salt-responsiveness (Figure 1, C), given that an ion transporter renders W05 more salt-tolerant (Qi et al., 2014). Under abiotic stresses, alternative splicing controls not only mRNA stability but also the active:inactive protein ratio governing interaction of some regulators with associates (Qin et al., 2017; Laloum et al., 2018).

This study exemplifies the latter mechanism. The splice variants of Class II *GmACBPs* encoded truncated proteins (Figure 1, D), which were ER-targeted resembling the native forms (Figure 2) and Class II ACBPs from Arabidopsis (Li and Chye, 2003; Lung et al., 2017) and rice (Meng et al., 2014; Liao et al., 2020). Pull-down experiments identified GST:ACBP4.1 interaction with seven type-1 LOXs (Figure 3, A–C). The one with the highest PSM (Figure 3, C), VLXB, was subject to further studies, revealing its colocalization (Figure 3, E and F) and interaction (Figure 4, A and Supplemental Figure S4) with native Class II GmACBPs at the ER. Immunocytochemistry has indicated cytosolic VLXB localization in paraveinal mesophyll cells (Stephenson et al., 1998; Fischer et al., 1999), whereas its occasional observation in the region with abundant ER has not been further addressed (Turner et al., 2011). Youn et al. (2006) also speculated from the VLXB structure that its N-terminal eight-stranded β-barrel domain may interact with membrane proteins, which are reported herein to be Class II GmACBPs (Figures 3, 4, 6). Similarly, the equivalent domain of mammalian 5-LOXs interacts with membrane-associated arachidonate-binding proteins (Mancini et al., 1993).

Soybean type-1 LOXs from seeds (GmLOX1–GmLOX3) and vegetative homologs (VLXA–VLXE) from leaves have been characterized (Kato et al. 1992; Fuller et al., 2001; Youn et al., 2006; Sellhorn et al., 2011). *VLXB* was responsive to wounding and methyl JA, rationalizing its relevance to stress signaling (Saravitz and Siedow, 1996). Transcriptomic analysis had shown salt-induced upregulation of *VLXB* peaking at 4 hpt in soybean roots (Liu et al., 2019, 2021), consistent with its upregulation as revealed from comparative proteomic data (Ji et al., 2016). Despite conservation of VLXA–VLXE (Fuller et al., 2001), they were classified by structural and enzyme kinetic features into subgroup 1 (VLXA, VLXB, and VLXE) and subgroup 2 (VLXC and VLXD) (Youn et al., 2006; Sellhorn et al., 2011). Among the seven GST:ACBP4.1-interacting LOXs, subgroup 1 members may exhibit stronger affinity given their higher PSM (Figure 3, C). Together with the upregulation of all subgroup 1 (but not subgroup 2) members in salt-stressed soybean leaves and roots (Ji et al., 2016), their cooperative role with Class II GmACBPs is affirmable.

Structural comparison revealed flexibility of the substrate entry site of VLXB, suggesting its higher degree of regiospecificity and stereoselectivity (Youn et al., 2006; Sellhorn et al., 2011). Enzyme assays confirmed VLXB-catalyzed oxygenation of linoleic and linolenic acids at C9 or C13 position (Kato et al., 1992; Fischer et al., 1999; Fuller et al., 2001; Sellhorn et al., 2011). The 9- and 13-hydroperoxides are then metabolized via at least seven branches of the plant oxylipin pathway (Wasternack and Song, 2017), some of which involve multifunctional LOXs (Liavonchanka and Feussner, 2006). Considering also the possible interaction of Class II GmACBPs with several LOXs (Figure 3, C), the regulation of this very first step intricately influences extraplastidial synthesis of many oxylipins. Hence, Class II GmACBP-overexpressing plants were monitored by MDA measurement to reflect their lipid peroxidation status (Figures 9, B, 10, A), similar to other studies on plant type-1 LOXs (Hwang and Hwang, 2010; Lόpez et al., 2011; Keunen et al., 2013; Hou et al., 2015; Lim et al., 2015). Under normal conditions, *GmACBP4.2*-transformed soybean hairy roots had higher MDA content and LOX activity than the VC, whereas *GmACBP4.1*-transformed hairy roots exhibited reciprocal phenotypes (Figure 9, B and C). Salinity induced lipid peroxidation (Figure 9, B) and LOX activity (Figure 9, C) in all genotypes. However, the higher upregulation of LOX activity in GmACBP4.2-overexpressors and its lower upregulation in GmACBP4.1-overexpressors than the VC (Figure 9, C) did not correlate well with comparable extent of MDA alteration (Figure 9, B). The latter may be attributed to the saturation of peroxidized lipids and nonenzymatic lipid peroxidation under osmotic stress (Keunen et al., 2013; Lim et al., 2015).

Similar patterns were also observed in Arabidopsis (Figure 10, A and B). The overexpression of Class II GmACBP variants boosted the MDA content under normal and salt stress conditions (Figure 10, A), resembling Arabidopsis overexpressing pepper (*C.* *annuum*) type-1 LOX1 under normal, drought and biotic stress conditions (Hwang and Hwang, 2010; Lim et al., 2015). Conversely, the reduced MDA content (Figure 10, A) and LOX activity (Figure 10, B) in overexpressors of the native forms were similar to the effects of *CaLOX1* silencing and *AtLOX1* mutation (Hwang and Hwang, 2010). The negative regulation of VLXB activity by Class II GmACBPs was further substantiated by in vitro LOX assays (Figure 9, D). The higher salt-induced upregulation of MDA (Figure 9, F) and LOX activity (Figure 9, G) is also in agreement with more pronounced alternative splicing of Class II *GmACBPs* in C08 than W05 roots (Figure 1, C and D). Therefore, salt-induced alternative splicing of Class II *GmACBPs* is considered an adaptive response to stimulate LOX catalysis.

### A dual mechanism triggers oxylipin signaling

GmACBP4.1_33–354_ bound with long-chain acyl-CoAs (Figure 5, A and Supplemental Table S1), of which polyunsaturated species facilitated VLXB interaction (Figure 6, B). In protein–lipid overlay assays, GmACBP4.1_33–354_ interacted only with PA but not 14 other neutral or polar lipids (Figure 5, B). GmACBP4.1_33–354_ exhibited specificity for different PA species (Figure 5, C), which competed with acyl-CoAs for protein binding (Figure 5, D). BiFC analysis also indicated no VLXB interaction with GmACBP4.1 mutants (Figure 4, D and Supplemental Figure S4, B), of which acyl-CoA (Supplemental Figure S6) but not PA (Figure 5, C and D) binding was abolished. Collectively, these data suggest that VLXB will dissociate from GmACBP4.1 if its ligand switches from C18:2/C18:3-CoAs to PA.

Time-resolved analysis did not indicate a dramatic change of total PA in salt-stressed soybean roots (Figure 9, H) and leaves (Liu et al., 2021). Im et al. (2012) had also observed that total PA of soybean seedlings rose by 70% after 5 min of NaCl treatment and dropped subsequently. The transient PA increase in Arabidopsis peaked at 10–30 min after NaCl (Yu et al., 2010) and ABA (Zhang et al., 2004, 2009) treatment. In contrast, the coordination of discrete molecular species with other effects, for example, alternative splicing (Figure 1), lipid peroxidation status (Figure 9, F) and LOX activity (Figure 9, G), implicated 32:0-PA as a salt-stress signal for Class II GmACBP-mediated response (Figure 9, I). Coincidently, it had relatively higher affinity to GmACBP4.1 among several synthetic PA species tested (Figure 5, B and C). While 32:0-PA was the sole species boosted at 1.5–6 hpt in C08 roots (Figure 9, I), the compositional change of PA in salt-tolerant W05 roots was less notable (Supplemental Figure S11). In fact, the compositional change of molecular species is more critical for mediating specific salt stress responses by targeting discrete PA-binding/responsive proteins, for example, 34:2-PA for a mitogen-activated protein kinase (Yu et al., 2010) and NADPH oxidases (Zhang et al., 2009), and 36:2-PA for protein phosphatase 2C (Zhang et al., 2004) and H^+^-ATPase (Zhang et al., 2006).

**Figure 11:**
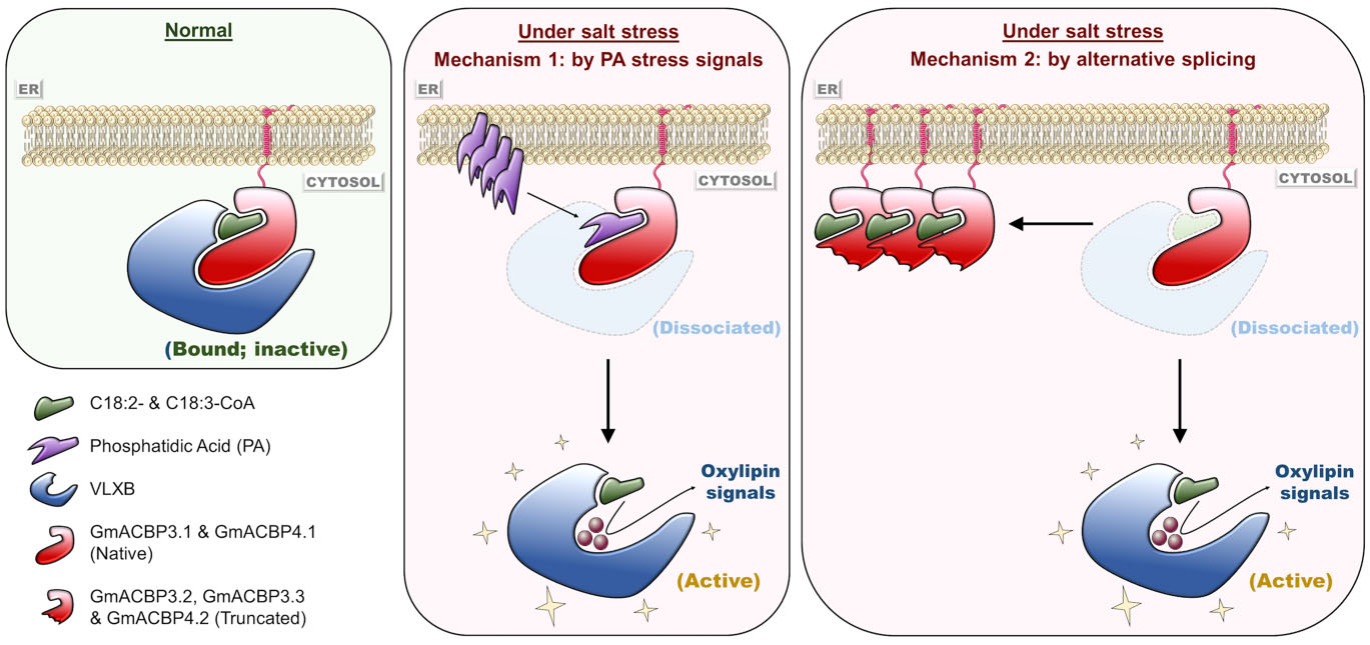
Model illustrating a dual mechanism for GmACBP3/GmACBP4-mediated modulation of oxylipin signaling in salinity response. Under normal conditions (left panel), GmACBP3.1 and GmACBP4.1 (native proteins) in complex with C18:2/C18:3-CoAs interact with VLXB, which is sequestered at the ER membrane, and potentially inactivated. Under salt stress (middle and right panels), binding of GmACBP3.1 and GmACBP4.1 with these acyl-CoAs is suppressed by a dual mechanism. First, specific PA species may act as a stress signal to compete for the ACB site of GmACBP3 and GmACBP4 (middle panel). Second, alternative splicing generates variants lacking the protein–protein-interacting domain (i.e. GmACBP3.2, GmACBP3.3, and GmACBP4.2), which compete with the native proteins for acyl-CoAs (right panel). Accordingly, unbinding of C18:2/C18:3-CoAs weakens GmACBP3.1 and GmACBP4.1 interaction with VLXB, which is liberated to generate oxylipin signals and trigger salinity response. In this process, the liberated C18:2/C18:3-CoAs, upon acyl-CoA thioesterase hydrolysis to form linoleic and linolenic acids, may also serve as substrates for VLXB (middle and right panels).

Hence, our findings unveil a dual mechanism that initiates the onset of oxylipin signaling in the salinity response via alternative splicing and PA signaling, as illustrated in Figure 11. Under normal conditions, C18:2/C18:3-CoA-liganded Class II GmACBPs inactivate VLXB by sequestering it at the ER (left panel). Under salt stress, these acyl-CoAs are competed out of protein binding in the presence of a PA signal (middle panel) and protein variants competing for the same ligands (right panel). VLXB is thereby liberated to trigger salt-responsive oxylipin synthesis (middle and right panels), as demonstrated by CoIP assays (Figure 10, C and D).

The activated VLXB may mediate a faster response if the co-liberated C18:2/C18:3-CoAs are converted concertedly by acyl-CoA thioesterase(s) into free PUFAs, the only substrate form for VLXB (Kato et al., 1992; Fuller et al., 2001; Sellhorn et al., 2011). This assumption is analogous to the mammalian system in which a membrane-localized arachidonate-binding protein facilitates substrate transfer to 5-LOX via protein–protein interaction (Mancini et al., 1993). In fact, the availability of free PUFAs is a rate-limiting factor for plant oxylipin synthesis (Wasternack and Feussner, 2018), as evident from the depletion of glycerolipid A_1_ lipases (Ishiguro et al., 2001; Wang et al., 2018). The stress-responsive role of such acyl-CoA thioesterase(s) has not been elucidated. Yet, it has been demonstrated that hypoxia-induced suppression of the reverse reaction catalyzed by acyl-CoA synthetase enriches unsaturated acyl-CoAs to modulate Class II AtACBPs (Schmidt et al., 2018; Zhou et al., 2020). While ER-localized acyl-CoA thioesterases (Tilton et al., 2000) and synthetases (Weng et al., 2010; Zhao et al., 2010) exist, their antagonistic coregulation for controlling the LOX substrate availability remains to be explored.

FA composition analysis indicated that salinity elevated PUFAs in *GmACBP4.2*/vector-transformed hairy roots and WT soybean roots, but not in the salt-hypersensitive *GmACBP4.1*-transformed hairy roots with higher C18:3 under normal conditions (Figure 9, A and E). The salt-induced enrichment of C18:2 and C18:3 is crucial for maintaining membrane integrity (Upchurch, 2008). After lipolytic release from acyl lipids, these PUFAs are the sole substrates for type-1 LOXs to synthesize oxylipins (Wasternack and Feussner, 2018). Consistently, Yu et al. (2020b) reported similar increase in C18:3 of most diacyl phospholipids after NaCl treatment of barley roots, more notably in salt-tolerant than salt-sensitive varieties. Contrary to soybean hairy roots, Arabidopsis overexpressing GmACBP3.1 and GmACBP4.1 did not exhibit an aberrant long-chain acyl-CoA composition under normal conditions (Supplemental Figure S10). It remains to be explored if inability to upregulate C18:3 is linked to the salt-hypersensitive phenotypes of plants overexpressing native Class II GmACBPs (Figures 7, 8). On the other hand, misregulation of lipid peroxidation (Figures 9, B, 10, A) and LOX activities (Figures 9, C, 10, B) appeared to be the casual factor in both soybean hairy roots and Arabidopsis.

### Similar oxylipin regulation in Arabidopsis

This work also implicates similar oxylipin regulation by Class II ACBPs in Arabidopsis. BiFC analysis indicated that AtLOX1, the closest homolog of VLXB, interacted with native Class II GmACBPs at the ER (Figure 4, B). Despite the lack of sorting or membrane-spanning peptides in VLXB (Supplemental Figure S3) and AtLOX1 (Melan et al., 1993), their DsRed-fusion proteins were ER-associated in Arabidopsis petioles and roots devoid of GmACBPs (Figure 3, F). This is attributed to their interaction with Class II AtACBPs, considering the 84% homology of the ANK domain between Class II AtACBPs and GmACBPs (Supplemental Figure S1), and the high expression of Class II *AtACBPs* in petioles and roots (Du et al., 2013a, 2013b). AtLOX1 has long been considered to reside in the cytosol (Melan et al., 1993; He et al., 2002), where 9-LOX reactions occur (Wasternack and Feussner, 2018). Subcellular proteomics also detected AtLOX1 in the endomembrane (Heard et al., 2015) and plasma membrane (Li et al., 2012; de Michele et al., 2016), matching the subcellular locations of Class II AtACBPs (Li and Chye, 2003; Lung et al., 2017). Surprisingly, AtLOX1:GFP was targeted to the chloroplast of Arabidopsis leaf protoplasts (Nalam et al., 2012), in line with the existence of a plastidial 9-LOX in rice (Zhou et al., 2014). Thus, AtLOX1 may be dually or differentially sorted depending on the cell type and status.

Overexpression of the native and variants forms of Class II GmACBPs in soybean hairy roots versus Arabidopsis produced similar effects, including the salinity phenotypes (Figures 7, 8), lipid peroxidation status (Figures 9, B, 10, A), and LOX activities in roots (Figures 9, C, 10, B). In fact, several clues support a similar dual mechanism for Class II AtACBP-modulated oxylipin signaling in the salinity response (Figure 11). First, C18:2/C18:3-CoAs are amongst the strongest ligands of AtACBP1 with 0.4–0.8 µM *K*_D_ values (Xue et al., 2014), close to that of GmACBP4.1 (0.9–1.2 µM; Supplemental Table S1). Resembling GmACBP4 (Figure 5, B–D), AtACBP1 is the sole isoform capable of PA binding (Du et al., 2010), of which the physiological relevance in signaling has been elucidated (Du et al., 2013a). Whereas *atacbp1* seedlings were more salt-tolerant than the WT, AtACBP1-overexpressing counterparts showed salt-hypersensitivity (Chen et al., 2018), similar to GmACBP3.1- and GmACBP4.1-overexpressing plants (Figures 7, 8). Transcriptomics also revealed Intron II retention of *AtACBP2* which was 2.3-fold upregulated under salinity (Feng et al., 2015), potentially truncating its ANK domain as in the salt-induced GmACBP3.3 (Figure 1, A–C).

Moreover, *AtLOX1* expression in roots was the highest of the six *AtLOXs* (Remans et al., 2010). *AtLOX1* was also stress-related as evident from its upregulation in senescing and *Pseudomonas syringae*-infiltrated leaves, as well as following ABA, methyl JA, and cadmium treatment in roots (Melan et al., 1993; He et al., 2002; Keunen et al., 2013). The overreaction of *lox1* to cadmium exposure (Keunen et al., 2013) and higher susceptibility of *lox1 lox5* to singlet oxygen and *P. syringae* infection (López et al., 2011) further substantiated the roles of type-1 AtLOXs in stress responses. Although type-1 9-LOXs are apparently irrelevant to JA biosynthesis that involves type-2 13-LOXs (Wasternack and Feussner, 2018), JA biosynthesis genes were suppressed in *lox1* roots, implicating crosstalk between 9-LOX and 13-LOX pathways in Arabidopsis (Keunen et al., 2013), as in rice (Zhou et al., 2014). More importantly, Class II *AtACBPs* (but not other *AtACBPs*) were downregulated in *lox1 lox5*, suggesting their functional relevance to type-1 AtLOXs (López et al., 2011).

### The ligand-binding status of ACBPs governs signaling

Although acyl-CoAs have long been regarded as intracellular signals in eukaryotes and some microbes (Neess et al., 2015; Lung and Chye, 2019), little is known regarding the molecular mechanism of action, for example, how does acyl-CoA binding to a regulatory protein mediate a downstream response? BiFC assays revealed that Ala substitutions at the ACB domain of GmACBP4 not only disrupted its binding with long-chain acyl-CoAs but also affected VLXB interaction (Figure 4, D and Supplemental Figure S4, B). Strep-Tactin pull-down assays further indicated that specific acyl-CoA species promoted different GmACBP4 interactions, that is,, C18:2/C18:3-CoAs for VLXB (Figure 6, B) versus C16:0/C18:1-CoAs for GmPLDα1 (Figure 6, C). These data agree with accumulating evidence that the ligand-binding status of ACB domain-containing proteins governs an allosteric macromolecular interaction site to switch on and off a response. In the archaeal model organism *Sulfolobus acidocaldarius*, the TetR-family transcription factor FadR_Sa_ negatively regulates a gene cluster for FA metabolism, whereas acyl-CoA liganding of FadR_Sa_ abrogates its DNA-binding domain thereby derepressing gene expression (Wang et al., 2019). The inhibitory effect of acyl-CoAs on FadR_Sa_–DNA complex formation varies with their acyl chain length (Wang et al., 2019). Comparatively, the unsaturation degree of acyl-CoAs is a determining factor for GmACBP4 interaction with VLXB (Figure 6, B) versus GmPLDα1 (Figure 6, C). The acyl-CoA responsiveness of FadR_Sa_ is desensitized by a protein kinase that phosphorylates the ACB pocket (Maklad et al., 2020), while acyl-CoA binding of GmACBP4 appears to be suppressed by a PA signal, that acts as a competitive ligand (Figure 5, D) upon its induction under stress (Figure 9, I).

Superimposition of the ligand- and DNA-bound structures of FadR_Sa_ revealed subtle differences that inferred the allosteric effect of acyl-CoA liganding on the DNA-binding domain (Wang et al., 2019). Analogously, docking of a specific ligand into the ACB pocket of GmACBP4 may structurally alter the ANK domain, favoring its interaction with certain interactors. Similarly, Class II AtACBP2, when complexed with lysophosphatidylcholine, exhibited 10-fold higher affinity for LYSOPL2 interaction via the ANK domain (Miao et al., 2019). Whilst structural biology of plant ACBPs is still in its infancy, we have resolved the first crystal structure of plant ACBPs using the prototypic Class I OsACBP1 and OsACBP2 (Guo et al., 2017; Jin et al., 2020). The structure of OsACBP2 liganded with C18:3-CoA deviates from its apo structure and the structure of prototypic human liver ACBP in complex with C14:0-CoA (Jin et al., 2020). The distinctive conformational features of an ACBP, when bound with discrete ligands, may govern the subset of its interactors, which may vary constantly with its changing ligand-binding status.

In plants, the mechanistic roles of acyl-CoAs have been deciphered solely in stress signaling and in concert with Class II ACBPs, that is, soybean GmACBP4 (this study) and Arabidopsis AtACBP1 and AtACBP2 (Schmidt et al., 2018; Zhou et al., 2020). Interestingly, long-chain unsaturated acyl-CoAs exert antagonistic effects by inhibiting oxylipin signaling (this study) while promoting hypoxia signaling (Schmidt et al., 2018; Zhou et al., 2020). C18:2/C18:3-CoAs facilitated GmACBP4–VLXB interaction (Figure 6, B) to suppress oxylipin synthesis (Figure 9, B and 10, A). In contrast, C18:1/C18:3-CoAs weakened AtACBP1/AtACBP2 binding with RAP2.12, and remobilized it from the plasma membrane into the nucleus for activating hypoxic genes, following hypoxia-induced polyunsaturation of long-chain acyl-CoAs (Schmidt et al., 2018; Zhou et al., 2020). Coincidentally, this work implicates that such an acyl-CoA composition change may have led to Class II ACBP-mediated suppression of oxylipin synthesis in hypoxia-stressed plants. This notion offers a tempting explanation for the decline in JAs and transcripts of JA biosynthesis genes including *AtLOXs* during prolonged submergence of Arabidopsis (Yuan et al., 2017). More intriguingly, upon postsubmergence reoxygenation of Arabidopsis, the reversion of C18:3-CoA back to its normal level (Zhou et al., 2020) may have desensitized interaction of Class II ACBPs with type-1 LOXs, accounting for the observed JA accumulation by Yuan et al. (2017). This potential oxylipin-related role of Class II ACBPs in hypoxic and posthypoxic stress responses remains to be addressed in future studies.

## Materials and methods

### Plant materials and growth conditions

Wild W05 (*G.* *soja*) and cultivated C08 (*G.* *max* [L.] Merr.) soybean accessions have been described previously (Lam et al., 2010). Soybean seeds were germinated in vermiculite at 25°C for 72 h. Seedlings were grown in half-strength Hoagland solution (Hoagland and Arnon, 1950) at 25–28°C under a 12-h light/12-h dark cycle. Plants with fully expanded primary leaves were treated in half-strength Hoagland solution with 0.9% (w/v) NaCl. For mRNA and protein analyses, all roots were harvested at 18:00 to minimize circadian effects. *Arabidopsis thaliana* (ecotype Columbia-0) seeds were stratified at 4°C stratification for 2 days, and germinated on Murashige and Skoog (MS) medium (Murashige and Skoog, 1962) plates with 1% (w/v) sucrose at 21°C under a long-day (16-h light/8-h dark) cycle. Arabidopsis was grown in MGRL solution (Fujiwara et al., 1992; Na^+^ excluded) according to Conn et al. (2013) at 21°C under a 10-h light/14-h dark cycle with 80% relative humidity and 100 μmol m^−2^ s^−1^ photon flux density. Four-week-old plants were treated in MGRL solution with 150 mM NaCl. Soybean and Arabidopsis tissues were frozen in liquid N_2_, homogenized to fine powder, and lyophilized for lipid peroxidation assays, LOX activity assays, and lipid analyses. *Nicotiana benthamiana* was grown at 25°C under a long-day cycle for 4 weeks prior to agroinfiltration.

### Plasmid construction for Arabidopsis transformation

All primers used in this study are listed in Supplemental Table S2. For OE constructs, *GmACBP3.1*, and *GmACBP4.1* open reading frames (ORFs) were amplified using primers ML3079/ML3128 and ML3079/ML3129, and cloned into pGEM-T Easy Vector (Promega, Madison, WI, USA) to generate plasmids pGM949 and pGM950, respectively. *Xba*I−*Xho*I fragments were excised from pGM949 and pGM950, and cloned into similar sites on pBI121-EGFP (Shi et al., 2005; replacing *EGFP*) to generate pGM951 and pGM952, respectively. *GmACBP3.3*, *GmACBP3.2*, and *GmACBP4.2* ORFs were amplified using primers ML3079/ML3163, ML3079/ML3164, and ML3079/ML3165, and cloned into *Xba*I−*Xho*I sites on pBI121-EGFP (replacing *EGFP*) to generate pGM955–pGM957, respectively. For *EGFP*-fusion constructs, *GmACBP3.1* and *GmACBP4.1* ORFs were amplified using primers ML3079/ML3080 and ML3079/ML3081, and cloned into pGEM-T Easy Vector to generate pGM943 and pGM944, respectively. *Xba*I–*Bam*HI fragments were excised from pGM943 and pGM944, and cloned into similar sites on pBI121-EGFP to generate pGM947 and pGM948, respectively. *GmACBP3.3*, *GmACBP3.2*, and *GmACBP4.2* ORFs were amplified using primers ML3079/ML3238, ML3079/M3239, and ML3079/ML3240, and cloned into *Xba*I–*Bam*HI sites on pBI121-EGFP to generate pGM960–pGM962, respectively. For *DsRed*-fusion constructs, *VLXB* and *AtLOX1* ORFs were amplified using primers ML3423/ML3424 and ML3425/ML3426, and cloned into the *Sma*I site on pAT765 (Chen et al., 2018) to generate pGM1024 and pAT1025, respectively. Arabidopsis was transformed with pGM947, pGM948, pGM951, pGM952, pGM955–pGM957, pGM960–pGM962, pGM1024, and pAT1025 by the floral dip method (Clough and Bent, 1998). Homozygous T_3_ plants were used for experiments.

### Generation of BiFC constructs

*Xba*I–*Bam*HI fragments encoding GmACBP3.1, GmACBP4.1, GmACBP3.3, GmACBP3.2, and GmACBP4.2 were excised from pGM947, pGM948, and pGM960−pGM962, and cloned into similar sites of pSPYNE-35S (Walter et al., 2004) to generate pGM992, pGM995, pGM994, pGM993, and pGM996, respectively. *GmACBP4.1* was mutated by overlap-extension PCR using mutagenic primers ML3413/ML3414 (Y135A), ML3415/ML3416 (K139A), ML3417/ML3418 (K161A), and ML3419/ML3420 (Y180A) with outermost primers ML3079/ML3081 (for all mutants). The mutated products were cloned into pGEM-T Easy Vector to generate pGM1008–pGM1011, from which *Xba*I–*Bam*HI fragments were excised and cloned into similar sites of pSPYNE-35S to generate pGM1012–pGM1015, respectively. *VLXB* and *AtLOX1* ORFs were amplified using primers ML3394/ML3395 and ML3400/ML3401, and cloned into pGEM-T Easy Vector to generate pGM1002 and pGM1003, respectively, from which *Xba*I–*Xho*I fragments were excised and cloned into similar sites on pSPYCE-35S (Walter et al., 2004) to generate pGM1006 and pAT1007, respectively.

### Confocal laser scanning microscopy

*Nicotiana* *benthamiana* leaves were infiltrated with *Agrobacterium tumefaciens* strain GV3101 harboring pGM947 and pGM948 for GmACBP3.1:EGFP and GmACBP4.1:EGFP expression, respectively. Their colocalization with mCherry-HDEL and DsRed:VLXB was verified by coinfiltration with *A. tumefaciens* harboring plasmids ER-rk (Nelson et al., 2007) and pGM1024, respectively. *Nicotiana* *benthamiana* and transgenic Arabidopsis expressing GmACBP3.1:EGFP (pGM947), GmACBP3.2:EGFP (pGM961), GmACBP3.3:EGFP (pGM960), GmACBP4.1:EGFP (pGM948), GmACBP4.2:EGFP (pGM962), DsRed:VLXB (pGM1024), and DsRed:AtLOX1 (pAT1025) were examined with a Zeiss LSM 710 NLO confocal laser scanning microscope. Arabidopsis seedlings were stained with 1 μM Invitrogen ER-Tracker Red (catalog no. E34250) and Green (catalog no. E34251), prior to two 5-min washes in deionized water, to confirm ER localization of EGFP- and DsRed-fusion proteins, respectively. For BiFC analysis, *N. benthamiana* leaves were coinfiltrated with *A. tumefaciens* harboring cYFP (pGM1006 or pAT1007) and nYFP (pGM992–pGM996; pGM1012–pGM1015) fusion constructs, together with an mCherry-HDEL marker to indicate successful transfection. Identical acquisition settings were kept for all BiFC analysis. Fluorescence was detected using the excitation and emission wavelengths: EGFP (488 nm/495–545 nm); YFP and ER-Tracker Green (514 nm/520–560 nm); DsRed and mCherry (543 nm/560–660 nm); ER-Tracker Red (543 nm/617–641 nm). Multiple fluorophores were scanned in a sequential mode to avoid spectral bleed-through. Colocalization analysis was performed using the Just Another Colocalization Plug-in for ImageJ (Bolte and Cordelières, 2006). Pearson’s correlation coefficient and Manders’ overlap coefficients M1 and M2 above Costes’ automatic thresholds were computed from eight-bit grayscale images after background subtraction with a rolling ball radius of 20 pixels.

### RT-PCR and qRT-PCR

Total RNAs were extracted using the RNeasy Plant Mini Kit (Qiagen, Hilden, Germany) and reverse-transcribed using the EvoScript Universal cDNA Master Kit (Roche, Basel, Switzerland) primed with oligo(dT). For RT-PCR, *ACBP3*, and *ACBP4* were amplified using primers ML3079/ML3128 under conditions: 95°C for 2 min, then 30 cycles of 95°C for 30 s, 60°C for 30 s, and 72°C for 90 s, with a final step of 72°C for 7 min. *ELF1b* was amplified using primers ML3349/ML3350 under conditions: 95°C for 2 min, then 28 cycles of 95°C for 30 s, 60°C for 30 s, and 72°C for 10 s, with a final step of 72°C for 7 min. The cycle numbers were within the predetermined linear amplification range. qRT-PCR was run in a CFX96 (Bio-Rad, Hercules, CA, USA) Real-Time PCR System using SYBR Premix Ex *Taq* reagents (Takara, Shiga, Japan) under conditions: 95°C for 30 s, then 40 cycles of 95°C for 5 s and 60°C for 30 s. Primers are listed in Supplemental Table S2. Relative expression was normalized against *ELF1b* using the comparative 2^−ΔCt^ method.

### Immunoblotting

Soybean roots were ground in 50 mM Tris–HCl (pH 8.0), 0.2 M NaCl, 1 mM EDTA and 1% (v/v) Triton X-100, and *N. benthamiana* leaves were homogenized in 50 mM Tris–HCl (pH 7.5), 0.15 M NaCl, 2 mM EDTA, 10% (v/v) glycerol, 0.2% (v/v) β-mercaptoethanol, 1% (v/v) Triton X-100, and 2% (w/v) polyvinylpolypyrrolidone, after addition of 1% (v/v) protease inhibitor cocktail (Sigma-Aldrich, St. Louis, MO, USA; catalog no. P9599) and 1 mM phenylmethylsulfonyl fluoride. Proteins were clarified by centrifugation at 20,000 *g* for 30 min. Subcellular fractionation of Arabidopsis shoots was performed following Smith et al. (1988). Proteins were resolved by 10% sodium dodecyl sulfate-polyacrylamide gel electrophoresis (SDS-PAGE), transferred to polyvinylidene difluoride membranes (Pall Life Sciences, New York, NY, USA; catalog no. BSP0161), and incubated with rabbit polyclonal antibodies (1:2,000) raised against (His)_6_-GmACBP3.1_33–346_, anti-nYFP (1:2,000; Agrisera, Vännäs, Sweden; catalog no. AS11-1776), or anti-cYFP antibodies (1:5,000; Agrisera; catalog no. AS11-1775) at 4°C overnight, and horseradish peroxidase-conjugated secondary antibodies (1:10,000; Sigma-Aldrich) at room temperature for 2 h. Signals were detected with an Alliance Q9 Advanced System (UVITEC) using the Pierce ECL Western Blotting Substrate (Thermo Scientific), and quantified densitometrically with the Gel Analyzer Tool of Image J version 1.4 software (National Institutes of Health). A linear calibration curve was constructed with 1–32 ng (His)_6_-GmACBP3.1_33–346_ per lane for measuring the level of Class II ACBPs in soybean roots.

### Recombinant protein expression and purification

For (His)_6_-tagged proteins, fragments encoding GmACBP3.1_33–346_ and GmACBP4.1_33–354_ were amplified using primers ML3150/ML3151 and ML3150/ML3129, and cloned into *Xho*I–*Eco*RI and *Xho*I sites of pRSETA (Invitrogen, Waltham, MA, USA) to generate pGM958 and pGM963, respectively. Fragments encoding GmACBP4.1_33–354_ Y135A, K139A, K161A, and Y180A mutants were amplified from pGM1008–pGM1011, and cloned into the *Xho*I site of pRSETA to generate pGM1020–pGM1023, respectively. For StrepII-tagged proteins, *VLXB* and *GmPLDα1* ORFs were amplified using primers ML3432/ML3433 and ML3297/ML3436, and cloned into *Xho*I and *Bam*HI–*Hin*dIII sites of pRSETA to generate pGM1027 and pGM1032, respectively. For GST-fusion proteins, a fragment encoding GmACBP3.1_33–346_ was amplified using primers ML3150/ML3128, and cloned into the *Xho*I site of pGEX-6P-1 (GE Healthcare, Chicago, IL, USA) to generate pGM970. An *Xho*I–*Xho*I fragment encoding GmACBP4.1_33–354_ was excised from pGM963, and cloned into the *Xho*I site of pGEX-6P-1 to generate pGM971. Recombinant proteins were expressed in *Escherichia coli* under specified conditions (Supplemental Figure S5C) following Miao et al. (2019). (His)_6_-tagged proteins were purified by immobilized metal affinity (HisTrap HP) and anion-exchange (HiTrap Q HP) chromatography columns (GE Healthcare) according to Guo et al. (2017). (His)_6_/StrepII-tagged proteins were purified on HisTrap HP columns (GE Healthcare) and Strep-Tactin XT resins (IBA), and GST-fusion proteins on GSTrap HP affinity and HiTrap Q columns (GE Healthcare), following the manufacturer’s instructions.

### Protein interactor identification

Purified GST and fusion proteins were immobilized to glutathione agarose at 4°C for 2 h using the Pierce GST Protein Interaction Pull-Down Kit (Thermo Scientific). The agarose was incubated with crude soybean soluble proteins at 4°C for 4 h. After five washes in Wash Buffer, bound proteins were eluted in Tris-buffered saline (TBS) with 10 mM glutathione, resolved by 10% SDS-PAGE, and visualized by silver staining. For protein identification, gel slices were reduced in 10 mM tris(2-carboxyethyl)phosphine, alkylated in 55 mM 2-chloroacetamide, and in-gel digested with 1 μg mL^−1^ trypsin at 37°C overnight. Tryptic peptides were extracted with 50% (v/v) acetonitrile in 5% (v/v) formic acid, and 100% (v/v) acetonitrile. After desalting with C18 StageTips, the peptides were analyzed by LC–MS/MS with a Dionex Ultimate3000 nanoRSLC system coupled to Thermo Fisher Orbitrap Fusion Tribid Lumos mass spectrometer at the Proteomics and Metabolomics Core Facility of the University of Hong Kong. Confident proteins were identified using a target–decoy approach with a reversed database, strict false-discovery rate of <1% at peptide and PSM levels.

### Isothermal titration calorimetry

ITC was conducted with a MicroCal iTC200 system (GE Healthcare) at 25°C in 10 mM sodium phosphate (pH 7) by 20 injections of 1.8-μL acyl-CoAs (200 μM; Avanti Polar Lipids Inc., Alabaster, AL, USA) into the sample cell with 15 μM purified (His)_6_-GmACBP4.1. Each injection lasted 4 s at 150-s intervals with a stirring speed of 1,000 rpm. Nonspecific heat effects were estimated and corrected after saturation. The baseline was adjusted, and the data were fitted into a single-site binding model using the Origin version 7.0 software (OriginLab).

### Lipid binding assays

Di-16:0-PC, di-14:0-PA, and di-16:0-PA were purchased from Echelon BioSciences, and di-18:0-PA and di-18:1-PA from Avanti Polar Lipids. Protein–lipid overlay assays were performed following Du et al. (2010). Commercial lipid filter strips (Echelon Biosciences, Salt Lake City, UT, USA; catalog no. P-6002) and nitrocellulose filter strips spotted with 5 μL of 6.25–50 μM di-14:0-, di16:0-, and di-18:0-PA were incubated with 0.5 μg mL^−1^ (His)_6_-GmACBP3.1_33–346_ or (His)_6_-GmACBP4.1_33–354_. Liposome association assays were modified from previous reports (Lu and Benning, 2009; Nakamura et al., 2014). Liposomes were prepared from PA (di-14:0, di-16:0, di-18:0, or di-18:1) and di-16:0-PC mixtures. After two washes in ice-cold TBS, 100 μg of liposomes were incubated with 5 μg mL^−1^ (His)_6_-GmACBP4.1_33–354_ in 100 μL of TBS at 30°C for 30 min. Protein–lipid bead binding assays (Rao et al., 1999; Nieto-Posadas et al., 2012) were performed by incubating 1 µM PA beads (Echelon Biosciences) with 2.5 µg mL^−1^ (His)_6_-GmACBP4.1_33–354_ and C18:2-CoA (0.3, 1, 3, 10, 30, 100, and 300 μM) in 50 μL of Binding Buffer containing 10 mM HEPES (pH 7.4), 0.15 M NaCl, 0.5% (v/v) Igepal CO-630 at 4°C for 3 h. After three washes in the same buffer, bound proteins were eluted with 6× Laemmli sample buffer. Protein binding on filter strips, liposomes, and PA beads was detected by immunoblotting using anti-ACBP3.1 antibodies.

### Strep-Tactin pull-down assays

Strep-Tactin pull-down assays were performed using Strep-Tactin XT resins (IBA) following the manufacturer’s instructions. Briefly, 5 μL of resins with/without prebound VLXB:StrepII or GmPLDα1:StrepII (0.5 μM) were agitated with GmACBP4.1_33–354_ (0.5 μM) and acyl-CoAs (0, 12.5, 25, 50, and 100 μM) in Buffer W containing 0.1 M Tris–HCl (pH 7), 0.15 M NaCl, 1 mM EDTA and 0.05% (w/v) Triton X-100 at 4°C for 2 h. After three washes in Buffer W, bound proteins were eluted in Buffer W with 50 mM biotin, and analyzed by immunoblotting using anti-ACBP3.1 antibodies.

### Coimmunoprecipitation

Double homozygous Arabidopsis lines expressing GmACBP4.1:EGFP and GmACBP4.2:EGFP with DsRed:VLXB were identified after genetic crosses. Roots were harvested from 4-week-old plants with/without 150 mM NaCl treatment for CoIP, as described previously (Lung et al., 2017). Briefly, 25 μL of Affi-Gel 10 beads (Bio-Rad) which had been coupled with anti-ACBP3 antibodies were incubated with 250 μL of 1 mg mL^−1^ crude protein extracts at 4°C for 1 h on a rotary shaker. The beads were washed thrice and eluted with 50 μL of elution buffer containing 0.1 M glycine-HCl (pH 2.4) and 0.15 M NaCl. The proteins were neutralized with 10 μL of 1 M Tris–HCl (pH 8), and analyzed by immunoblotting using anti-ACBP3 and anti-DsRed (1:2,000; Takara; catalog no. 632496) antibodies.

### Salt tolerance test of soybean hairy roots

*GmACBP4.1* and *GmACBP4.2* ORFs were amplified using primers ML3293/ML3129 and ML3293/ML3165, and cloned into the *Xho*I site of pCambia3301 (CAMBIA; replacing *bar*) to generate pGM973 and pGM974, respectively. *Agrobacterium rhizogenes* strain K599 (Kereszt et al., 2007) harboring pGM973, pGM974, or empty vector was grown in yeast extract peptone broth containing 50 μg mL^−1^ kanamycin, 50 μg mL^−1^ rifamycin, and 200 μM acetosyringone at 28°C for 20–24 h. The cells were pelleted at 5,000 rpm for 15 min, washed twice in 10 mM MgSO_4_, and adjusted to OD_600_ ≈ 0.5 for cotyledon infection. Soybean seeds (C08 cultivar) were germinated on moist paper towel at 28°C in the dark for 3 days. Five-day-old seedlings were grown hydroponically until primary leaf emergence. Cotyledons were scalpel-wounded, and infected with *A. rhizogenes* following Wei et al. (2016). Hairy roots were cultured in half-strength Hoagland solution with/without 100 mM NaCl for 10 days, prior to measurement of maximum length and fresh weight of roots and PCR verification for the presence of the transgene.

### Salt tolerance test of Arabidopsis

Arabidopsis seeds were germinated on MS plates. Four-day-old seedlings were transferred to fresh MS plates with/without 150 mM NaCl. Survival rate was calculated as the percentage of surviving plants after 14 days. Primary root growth was measured on vertically-oriented plates over an 8-day period. Four-week-old hydroponically-grown plants were examined after 10-day treatment in MGRL solution with/without 150 mM NaCl. Soil plant analysis development (SPAD) values for chlorophyll concentrations were determined at the center of five leaves per plant using a SPAD-502 Plus meter (Konica Minolta, Tokyo, Japan). For electrolyte leakage analysis, shoots and roots were agitated in 50 mL of deionized water at 23°C for 1 h. Initial conductivity was measured with a YSI Pro2030 conductometer. After boiling and cooling down to 23°C, total conductivity of the solution was measured again. Relative electrolyte leakage was calculated as the percentage of the initial over total conductivities. Water content was calculated as the weight loss after oven-drying at 80°C for 2 days on fresh weight basis. Na^+^ and K^+^ contents were measured according to Munns et al. (2010). Briefly, roots were rinsed with two 10-s dips in 10 mM Ca(NO_3_)_2_ and five times in deionized water. Shoots and roots were oven-dried at 80°C for 2 days. Powdered samples (8 mg mL^−1^) were digested with 0.5 M nitric acid at 80°C for 1 h, and clarified by centrifugation at 20,000 *g* for 15 min. Na^+^ and K^+^ contents were quantified with an atomic absorption spectrometer (iCE 3300; Thermo Scientific) using Na (0–3 ppm) and K (0–4 ppm) standard solutions (Sigma-Aldrich).

### FA and PA analyses

FAs were extracted from several mg of lyophilized powder as described by Guo et al. (2019b). FA methyl esters were analyzed with an Agilent GC–MS device (5973 inert mass spectrometer combined with 6890N gas chromatograph) equipped with an Agilent J&W DB-WAX capillary column (30 m × 0.25 mm × 0.25 µm) in a splitless mode. The temperature was set at 150°C for 3 min, then increased to 240°C at 10°C min^−1^, and kept at 240°C for 5 min. For PA analysis, 5–10 mg of lyophilized powder was treated with 0.01% (w/v) butylated hydroxytoluene in isopropanol (100 µL mg^−1^) at 75°C for 18 min to deactivate lipid-hydrolyzing enzymes, and extracted with chloroform:methanol:300 mM ammonium acetate (30:41.5:3.5; 300 µL mg^−1^) at 4°C for 30 min. The samples were clarified by centrifugation at 12,000 rpm, 4°C for 5 min. After another round of extraction, the supernatants were combined and dried under N_2_ gas. The extracted lipids were subject to normal phase analysis at LipidALL Technologies Company Ltd. (Changzhou, China) using an Exion UPLC-QTRAP 6500 Plus (Sciex) LC–MS/MS equipped with a Phenomenex Luna 3 µm silica column (internal diameter 150 × 2 mm) as reported previously (Liu et al., 2020). Individual PA species were quantified by reference to the spiked internal standards d7-PA33:1(15:0/18:1) and DMPA.

### Lipid peroxidation and LOX activity assays

Lipid peroxidation was analyzed by measuring the MDA content (Heath and Packer, 1968). Lyophilized powder was vortexed vigorously in a solution (100 µL mg^−1^) containing 10% (w/v) trichloroacetic acid, 0.5% (w/v) thiobarbituric acid, and 0.01% (w/v) butylated hydroxytoluene. The samples were heated at 95°C for 25 min, and cooled on ice for 5 min. After centrifugation at 13,000 *g* for 10 min, the absorbance of the supernatant was measured at 532 nm and corrected for nonspecific turbidity by subtracting the value at 600 nm. The MDA content was calculated from an extinction coefficient of 155 mM^−1^ cm^−1^. For LOX activity assays, lyophilized powder was vortexed vigorously at 4°C in a solution (100 µL mg^−1^) containing 100 mM sodium phosphate (pH 6.8) and 4% (w/v) polyvinylpolypyrrolidone. After centrifugation at 15,000 *g*, 4°C for 25 min, protein concentration of the supernatant was determined using the bicinchoninic acid assay kit (Thermo Scientific). Nonspecific activities of other enzymes (e.g. peroxidases, cytochromes P450) were eliminated by incubation with 1 mM KCN at 25°C for 10 min, which did not affect the activity of VLXB (Supplemental Figure S12). The LOX activity was assayed in 100 mM sodium phosphate (pH 6.8) at 25°C for 10 min with 0.5 mM linoleic acid (Sigma-Aldrich; catalog no. L1376), of which a 25 mM stock was prepared following Anthon and Barrett (2001). The conjugated diene products were measured spectrophotometrically by the absorbance increase at 234 nm using a molar extinction coefficient of 25,000 M^−1^ cm^−1^ (Axelroad et al., 1981). To study the inhibitory effect of GmACBP4.1 on VLXB, 100 nM VLXB:StrepII was incubated with 0.1–300 nM (His)_6_-GmACBP4.1_33–354_ and 10 µM acyl-CoA in 100 mM sodium phosphate (pH 6.8) at 25°C for 10 min, prior to LOX activity assays.

### Phylogenetic analysis

Protein sequences of LOX homologs were aligned using the MUSCLE algorithm with default parameters (gap open penalty, −2.9; gap extension penalty, 0; hydrophobicity multiplier, 1.2; clustering method, UPGMA), and a phylogenetic tree was constructed by the neighbor-joining method using the Molecular Evolutionary Genetics Analysis version 7.0 program. The alignment used for phylogenetic analysis is available as Supplemental File S1. The machine-readable phylogenetic tree file is provided in Newick format as Supplemental File S2. The reliability for internal branch was assessed using the bootstrapping method (2,000 bootstrap replicates).

### Statistical analysis

Statistical analysis was performed using the two-tailed Student’s *t* test. The results are shown in Supplemental Data Set S1.

## Accession numbers

Sequence data from this article can be found in the Phytozome (soybean) and TAIR (Arabidopsis) databases under accession numbers: ELF1b (Glyma.02G276600), GmPLDα1 (Glyma.07G031100), AtACBP1 (AT5G53470), AtACBP2 (AT4G27780), GmACBP3 (Glyma.04G233600), GmACBP4 (Glyma.06G131200), and LOXs (see Figure 3, D).

## Supplemental data

The following materials are available in the online version of this article.

**Supplemental Figure S1.** Amino acid sequence alignment of Class II ACBPs from Arabidopsis and soybean.

**Supplemental Figure S2.** Alignment of the nucleotide sequences encoding Class II ACBP3 and ACBP4 in wild W05 and cultivated C08 soybean.

**Supplemental Figure S3.** Prediction of the subcellular location and putative TM domain of Class II GmACBPs and GmACBP4.1-interacting LOXs.

**Supplemental Figure S4.** GmACBP3 and GmACBP4 splice variants and GmACBP4.1 mutants did not interact with VLXB in BiFC assays.

**Supplemental Figure S5.** Expression and purification of recombinant proteins.

**Supplemental Figure S6.** Effect of single amino acid substitutions on GmACBP4.1 interaction with acyl-CoAs.

**Supplemental Figure S7.** Strep-Tactin pull-down assays for the interaction of GmACBP4.1 mutants with VLXB in the presence of acyl-CoAs.

**Supplemental Figure S8.** PCR verification of transgenic soybean hairy roots.

**Supplemental Figure S9.** Salt tolerance test and mock treatment of transgenic Arabidopsis expressing native form and splice variants of Class II GmACBPs.

**Supplemental Figure S10.** FA composition of transgenic Arabidopsis expressing Class II GmACBPs.

**Supplemental Figure S11.** Molecular species composition of PA in soybean roots under salinity.

**Supplemental Figure S12.** In vitro enzyme activity of VLXB in the presence of potassium cyanide.

**Supplemental Table S1.** Binding constants and thermodynamic parameters for (His)_6_-GmACBP4.1_33–354_ interaction with acyl-CoAs.

**Supplemental Table S2.** Sequences of primers used in this study.

**Supplemental File S1.** Amino acid alignment of soybean and Arabidopsis LOX homologs.

**Supplemental File S2.** LOX phylogenetic tree in Newick format.

**Supplemental Data Set S1.** Statistical analysis results.

## Supplementary Material

koab306_Supplementary_Data

## Notes

S.-C.L., S.H.L., H.W., X.Z., A.L., and Z.-H.G. performed the research. S.-C.L., S.H.L., H.W., X.Z., A.L., Z.-H.G., H.-M.L., and M.-L.C. analyzed the data. S.-C.L. and M.-L.C. designed the research and wrote the manuscript with contributions from all authors. H.-M.L. and M.-L.C. acquired funding for the research.

The author responsible for distribution of materials integral to the findings presented in this article in accordance with the policy described in the Instructions for Authors (https://academic.oup.com/plcell) is: Mee-Len Chye (mlchye@hku.hk).
