# Supplementary material for: Oxylipin signaling in salt-stressed soybean is modulated by ligand-dependent interaction of Class II acyl-CoA-binding proteins with lipoxygenase
Source: Plant Cell. 2021 Dec 17;34(3):1117–43. doi: 10.1093/plcell/koab306 (PMC8894927; doi:10.1093/plcell/koab306)
Supplement: koab306_Supplementary_Data [file koab306_supplementary_data.zip › tpc.21.00410_SupplementalFiguresandTables 12721.pdf]

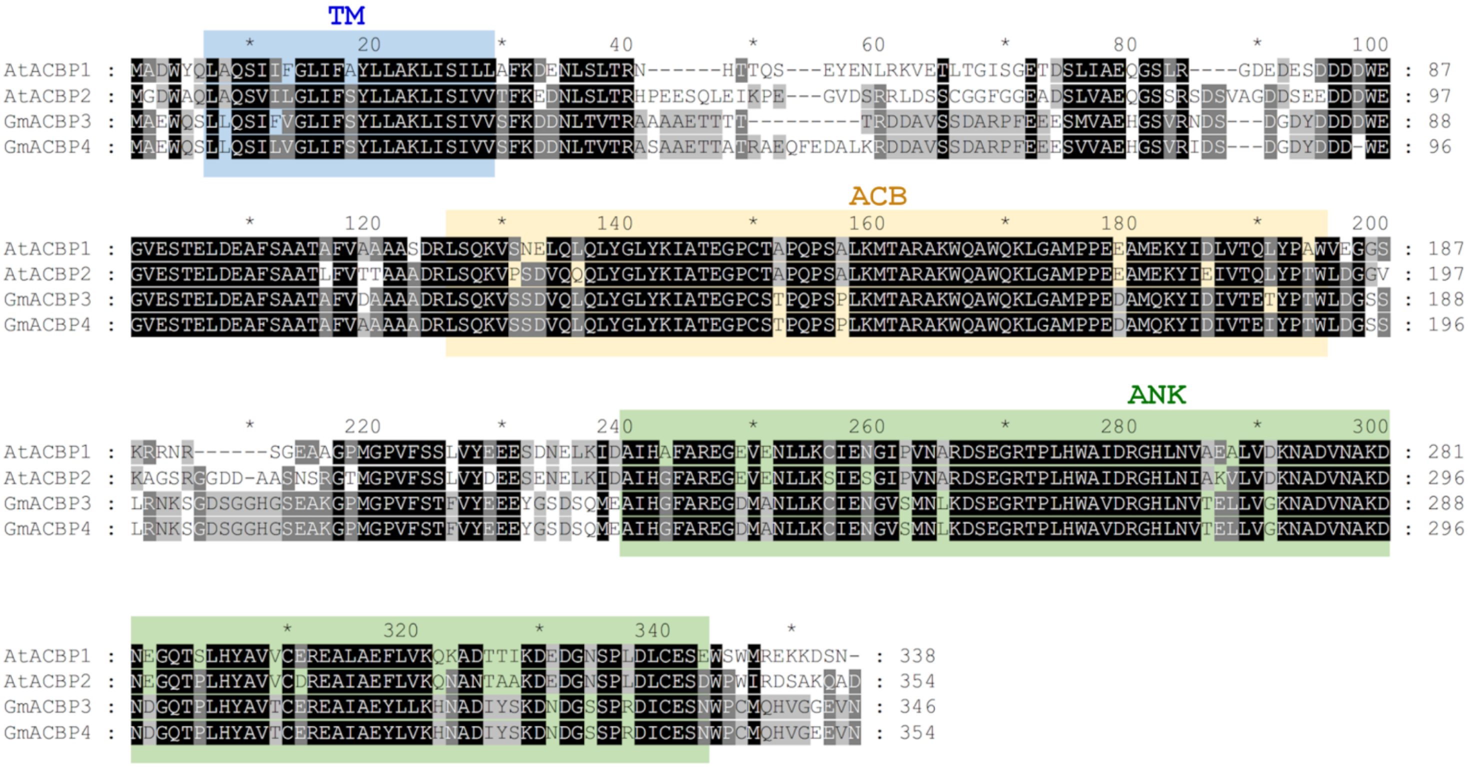

Supplemental Figure 1. Amino acid sequence alignment of Class II ACBPs from Arabidopsis and soybean. (Supports Figure 1A)

Amino acid sequences of Class II ACBPs from Arabidopsis (*Arabidopsis thaliana*) and soybean (*Glycine max*) were aligned using Clustal W v1.83. Residue background colors: black (100% conserved), dark gray (≥75% conserved) and light gray (50–74% conserved). The transmembrane (TM, blue), acyl-CoA-binding (ACB, tan) and ankyrin-repeat (ANK, green) domains are highlighted.

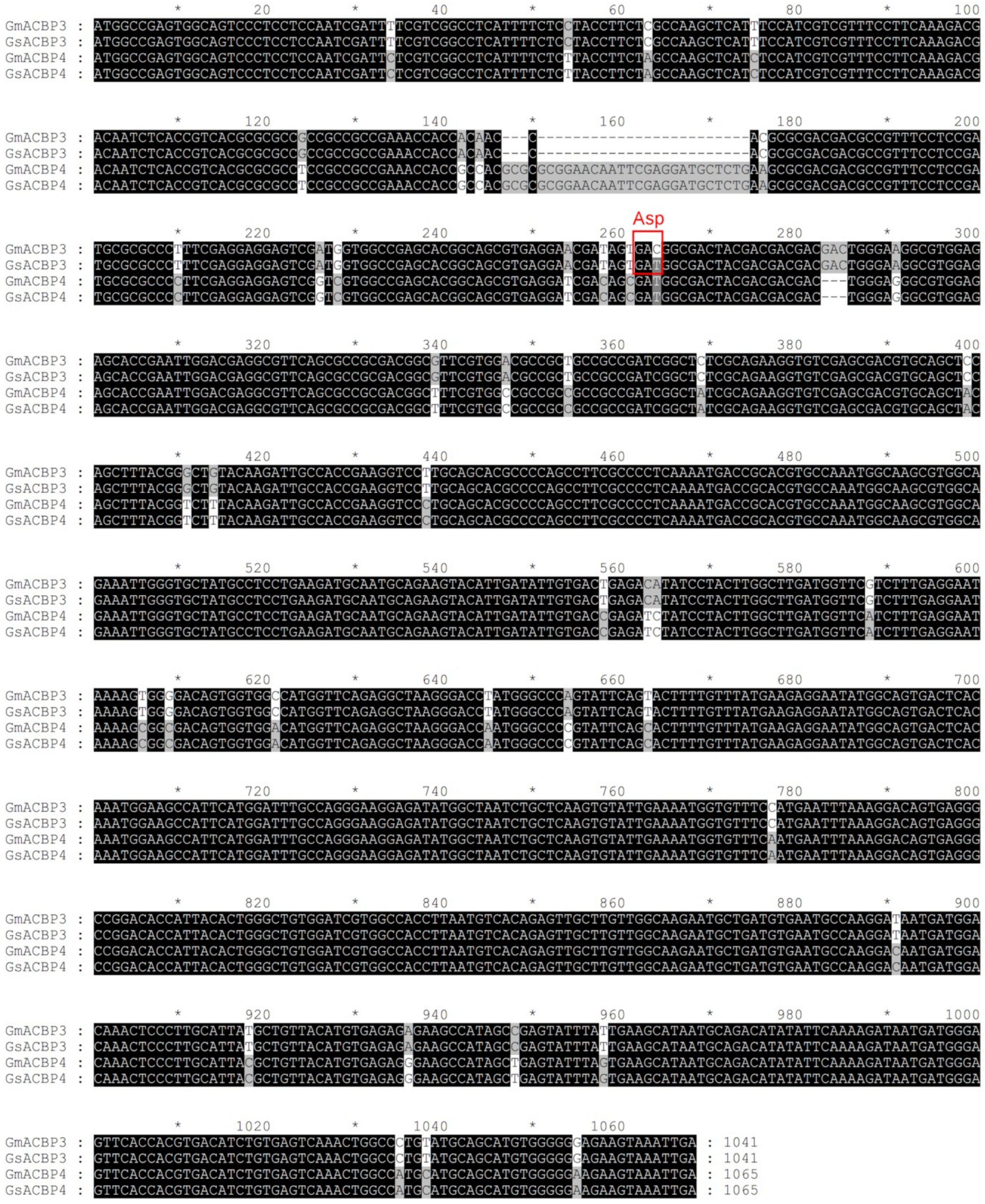

Supplemental Figure 2. Alignment of the nucleotide sequences encoding Class II ACBP3 and ACBP4 in wild W05 and cultivated C08 soybean. (Supports Figure 1A)

Sequences were aligned using Clustal W v1.83. Nucleotide background colors: black (identical), dark gray (75% conserved) and light gray (50% conserved). A single nucleotide polymorphism of *ACBP3* (boxed in red) from wild W05 soybean (*Glycine soja*) vs cultivated C08 soybean (*Glycine max*) resulted in different codons for Asp-79.

Supplemental Data. Lung et al. (2022). Plant Cell. Oxylipin signaling in salt-stressed soybean is modulated by ligand-dependent interaction of Class II acyl-CoA-binding proteins with lipoxygenase.

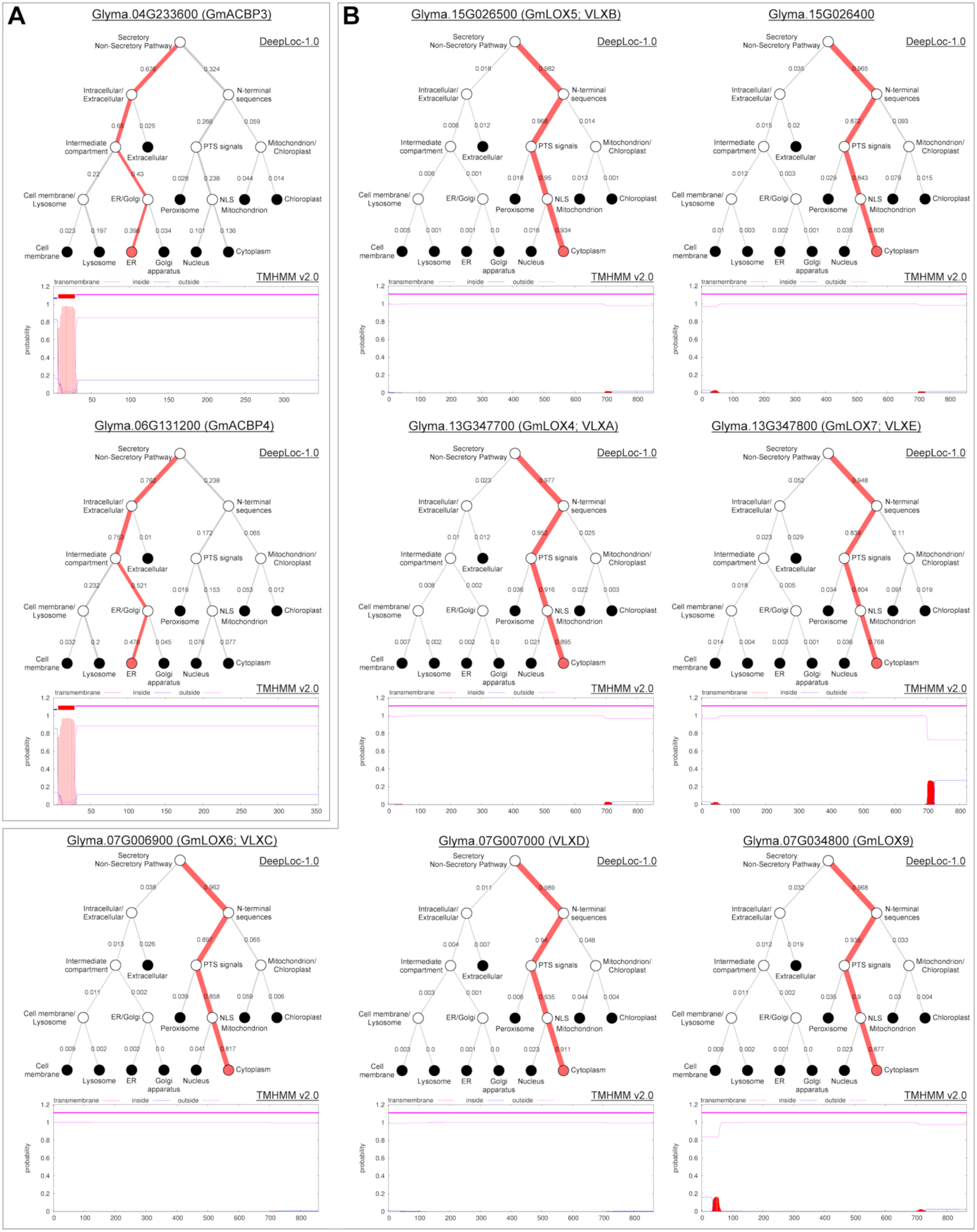

Supplemental Figure 3. Prediction of the subcellular location and putative transmembrane domain of Class II GmACBPs and GmACBP4.1-interacting LOXs. (Supports Figures 2, 3E and 3F)

The subcellular location was predicted by using the eukaryotic protein subcellular localization predictor based on the deep neural network algorithm, DeepLoc-1.0 (http://www.cbs.dtu.dk/services/DeepLoc/index.php; Almagro Armenteros et al., 2017). The red zigzag lines indicate the most probable path of protein sorting to the various subcellular destinations (black circles). The putative transmembrane domain was predicted by using the TMHMM server v2.0 (http://www.cbs.dtu.dk/services/TMHMM/).

(A) Class II GmACBPs were predicted to be ER-associated proteins with an *N*-terminal transmembrane domain.

(B) The seven GmACBP4.1-interacting LOXs were predicted to be cytosolic proteins devoid of any transmembrane domain.

Reference: Almagro Armenteros, J.J., Sønderby, C.K., Sønderby, S.K., Nielsen, H., and Winther, O. (2017). DeepLoc: prediction of protein subcellular localization using deep learning. Bioinformatics **33:** 3387–3395.

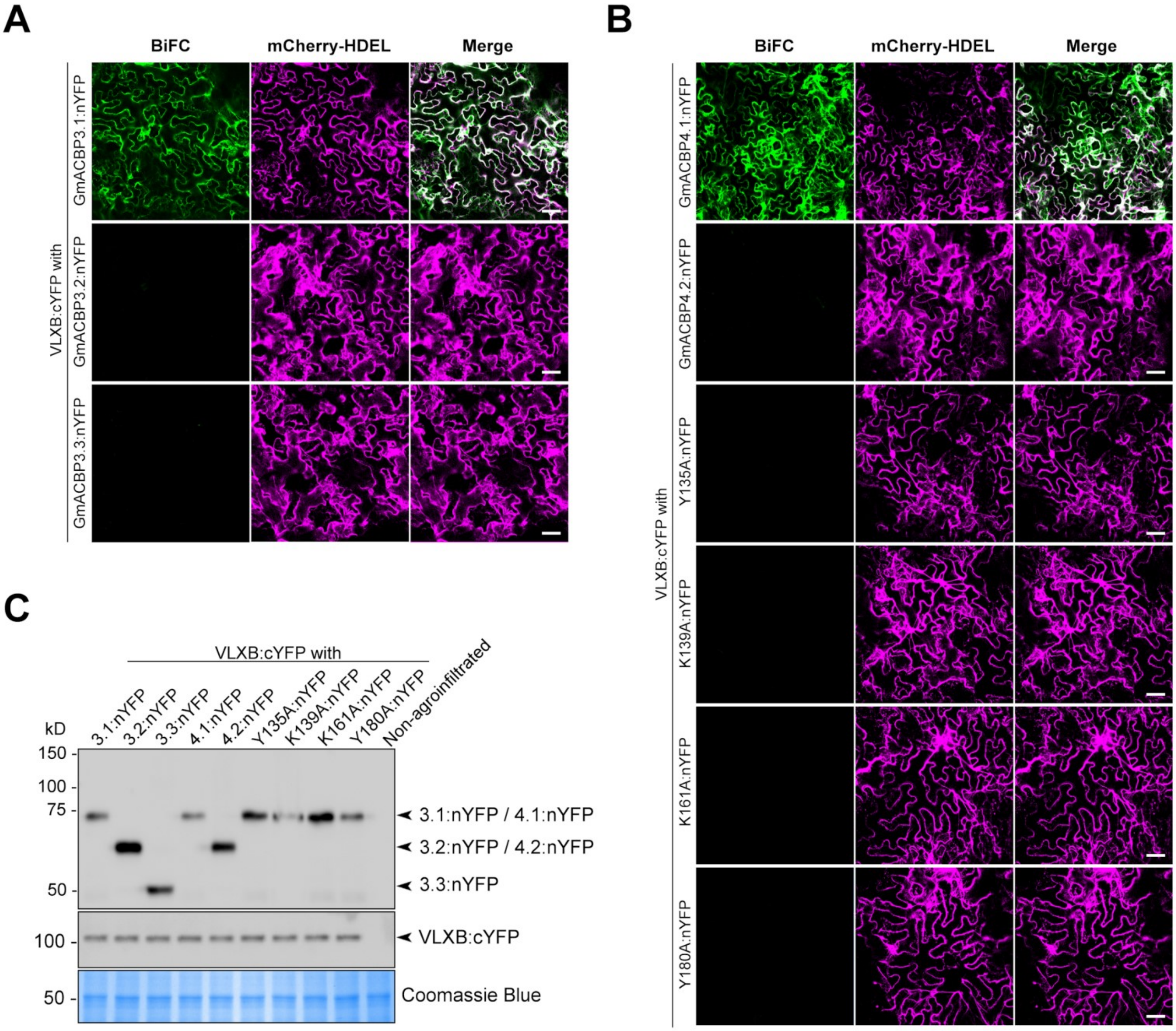

Supplemental Figure 4. GmACBP3 and GmACBP4 splice variants and GmACBP4.1 mutants did not interact with VLXB in bimolecular fluorescence complementation assays. (Supports Figures 4A and 4D)

Nicotiana benthamiana leaf epidermal cells were agroinfiltrated with split-YFP (nYFP & cYFP) fusion constructs together with the mCherry-HDEL ER marker as a transfection control. Bimolecular fluorescence complementation (BiFC) and mCherry-HDEL signals were examined by confocal laser scanning microscopy. Bars = 50 µm.

(A) VLXB:cYFP was transfected with nYFP-fusion proteins of native GmACBP3.1 and splice variants (GmACBP3.2 & GmACBP3.3).

(B) VLXB:cYFP was transfected with nYFP-fusion proteins of native GmACBP4.1, splice variant (GmACBP4.2), and GmACBP4.1 mutants (Y135A, K139A, K161A & Y180A).

(C) Immunoblot analysis of BiFC construct combinations from the same experiments as in **(A–B)**. Total proteins (15 µg/lane) were resolved on 10% SDS-PAGE and analyzed by immunoblotting with anti-nYFP (upper panel) and anti-cYFP antibodies (middle panel) and Coomassie Brilliant Blue staining (lower panel). Arrowheads indicate target bands of 58-kD GmACBP3.1:nYFP (apparent: 72 kD), 54-kD GmACBP3.2:nYFP (apparent: 65 kD), 42-kD GmACBP3.3:nYFP (apparent: 50 kD), 59-kD GmACBP4.1:nYFP (apparent: 72 kD), 55-kD GmACBP4.2:nYFP (apparent: 65 kD), and 108-kD VLXB:cYFP. Representative blots from three independent experiments are shown.

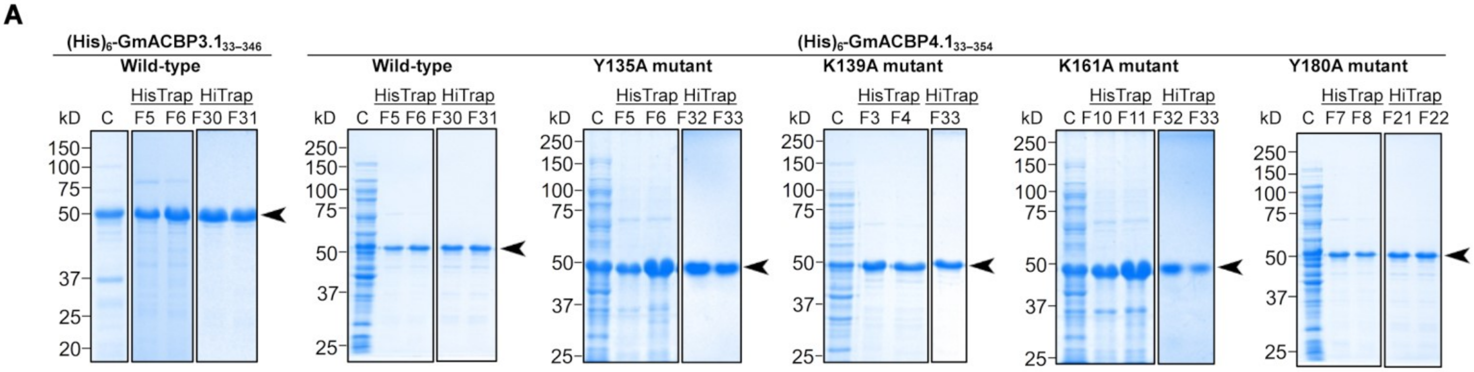

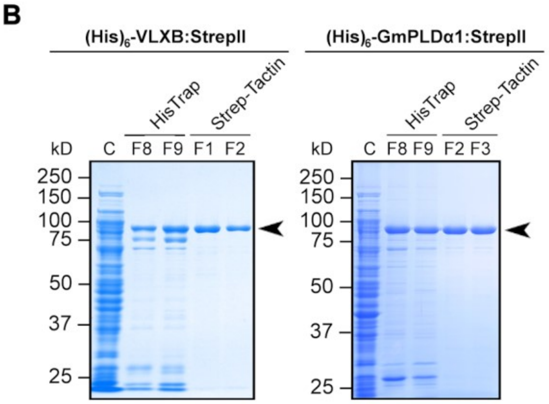

C

Recombinant protein	Plasmid	*Escherichia coli* host	Induction conditions		
			[IPTG]	Temperature	Duration
$(His)_6$-GmACBP3.1$_{33–346}$	pGM958	BL21 (DE3)	1 mM	37°C	3 h
$(His)_6$-GmACBP4.1$_{33–354}$	pGM963	BL21 (DE3)	0.2 mM	37°C	3 h
$(His)_6$-GmACBP4.1$_{33–354}$ -Y135A	pGM1020	BL21 (DE3) pLysS	1 mM	37°C	3 h
$(His)_6$-GmACBP4.1$_{33–354}$ -K139A	pGM1021	BL21 (DE3) pLysS	1 mM	37°C	3 h
$(His)_6$-GmACBP4.1$_{33–354}$-K161A	pGM1022	BL21 (DE3) pLysS	1 mM	37°C	3 h
$(His)_6$-GmACBP4.1$_{33–354}$-Y180A	pGM1023	BL21 (DE3) pLysS	0.2 mM	37°C	3 h
$(His)_6$-VLXB:StrepII	pGM1027	BL21 (DE3) pLysS	0.2 mM	23°C	16 h
$(His)_6$-GmPLDα1:StrepII	pGM1032	Rosetta2 (DE3)	0.2 mM	23°C	16 h
GST	pGEX-6P-1	BL21 (DE3)	0.5 mM	37°C	3 h
GST:GmACBP3.1$_{33–346}$	pGM970	Rosetta (DE3)	0.2 mM	37°C	3 h
GST:GmACBP4.1$_{33–354}$	pGM971	BL21 (DE3) pLysS	0.2 mM	37°C	3 h

Supplemental Figure 5. Expression and purification of recombinant proteins. (Supports Figures 5, 6 and 9D)

(A) Expression and purification of $(His)_6$-tagged GmACBP3.1$_{33–346}$ and GmACBP4.1$_{33–354}$ lacking the *N*-terminal transmembrane domain. Recombinant proteins were expressed in *Escherichia coli* and purified from soluble fractions on HisTrap HP immobilized metal affinity and HiTrap Q HP anion-exchange chromatography columns. Arrowheads indicate target bands of 39 kD $(His)_6$-GmACBP3.1$_{33–346}$ (apparent: 50 kD) and 40 kD $(His)_6$-GmACBP4.1$_{33–354}$ (apparent: 50 kD).

(B) Expression and purification of $(His)_6$/StrepII-tagged VLXB and GmPLDα1. Recombinant proteins were expressed in *E. coli* and purified from soluble fractions on HisTrap HP immobilized metal affinity chromatography columns and subsequently on Strep-Tactin resins. Representative Coomassie Blue-stained SDS-PAGE gels show proteins from total crude extracts (C) and eluted fractions (F). Arrowheads indicate target bands. Arrowheads indicate target bands of 102 kD $(His)_6$-VLXB:StrepII and 97-kD $(His)_6$-GmPLDα1:StrepII.

(C) Summary of *E. coli* host strains and conditions for recombinant protein expression by isopropyl *β*-D-1-thiogalactopyranoside (IPTG) induction.

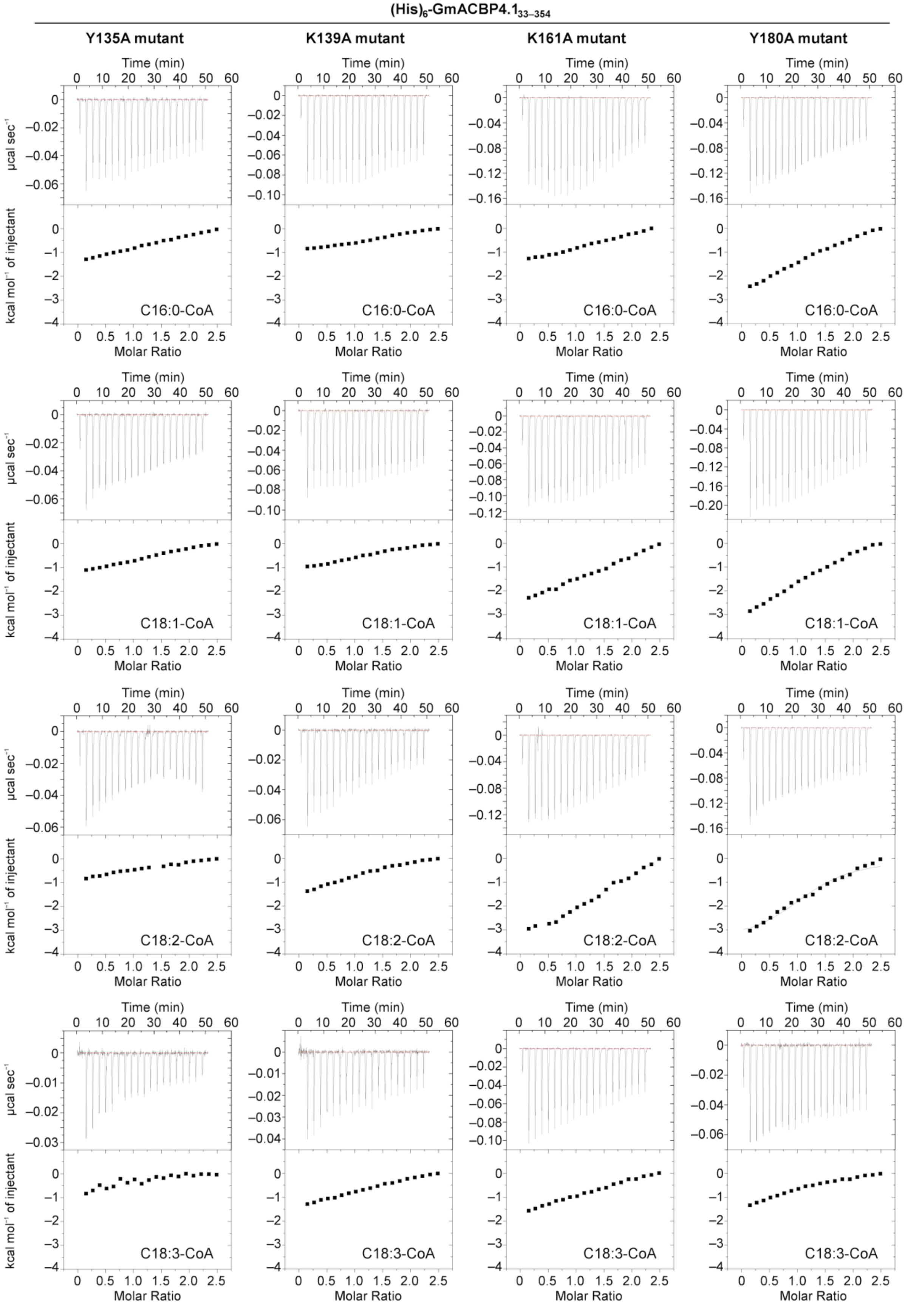

Supplemental Figure 6. Effect of single amino acid substitutions on GmACBP4.1 interaction with acyl-CoAs. (Supports Figure 5A)

Isothermal titration calorimetry was performed by titration of 250 µL of 15 µM (His)$_6$-GmACBP4.1$_{33–354}$ with 20 injections of 1.8-µL aliquots of 200 µM C16:0-, C18:1-, C18:2- or C18:3-CoAs at 25°C. Representative binding isotherms including raw heating power over time (upper panels) and integrated area of each injection after background correction (lower panels) are shown after similar results were obtained from two to four independent experiments.

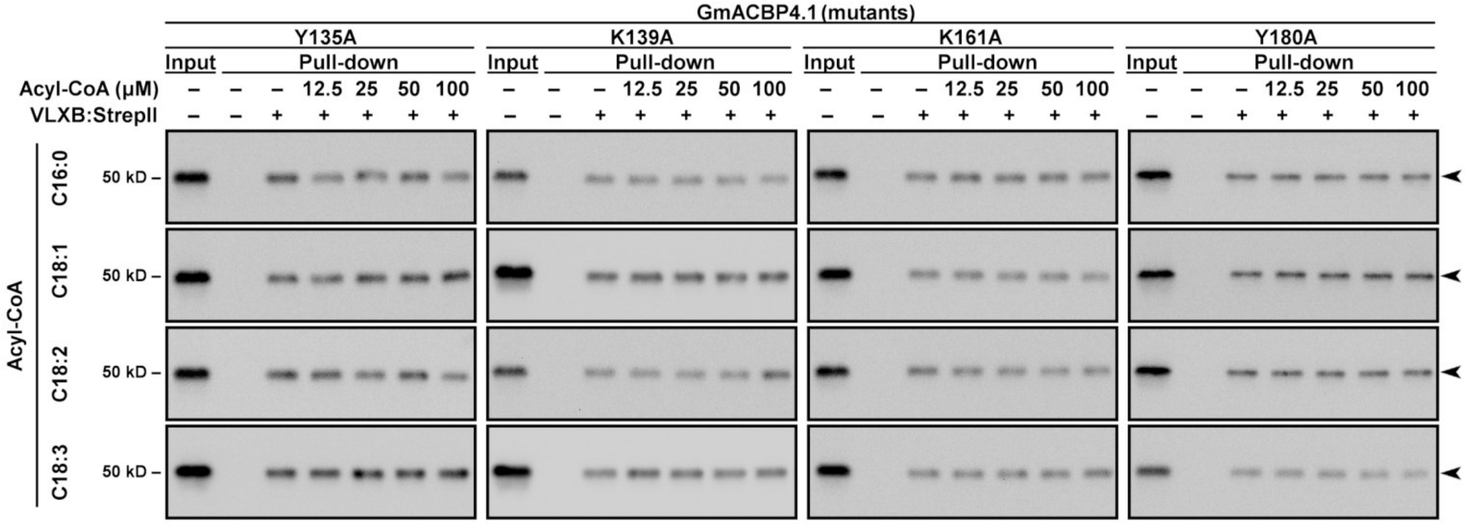

Supplemental Figure 7. Strep-Tactin pull-down assays for the interaction of GmACBP4.1 mutants with VLXB in the presence of acyl-CoAs. (Supports Figure 6B)

Strep-Tactin beads with/without pre-bound VLXB:StrepII were incubated with equimolar concentration of $(His)_6$-GmACBP4.$1_{33–354}$ (Y135A, K139A, K161A and Y180A mutants) in the presence of serial dilutions of C16:0-, C18:1-, C18:2 or C18:3-CoA. $(His)_6$-GmACBP4.$1_{33–354}$ in eluates were detected by immunoblotting using anti-ACBP3 antibodies. Representative blots are shown after similar results were obtained from at least two independent experiments. Input lanes represent 5% proteins. Arrowheads indicate target bands of 40-kD $(His)_6$-GmACBP4.$1_{33–354}$ (apparent: 50 kD).

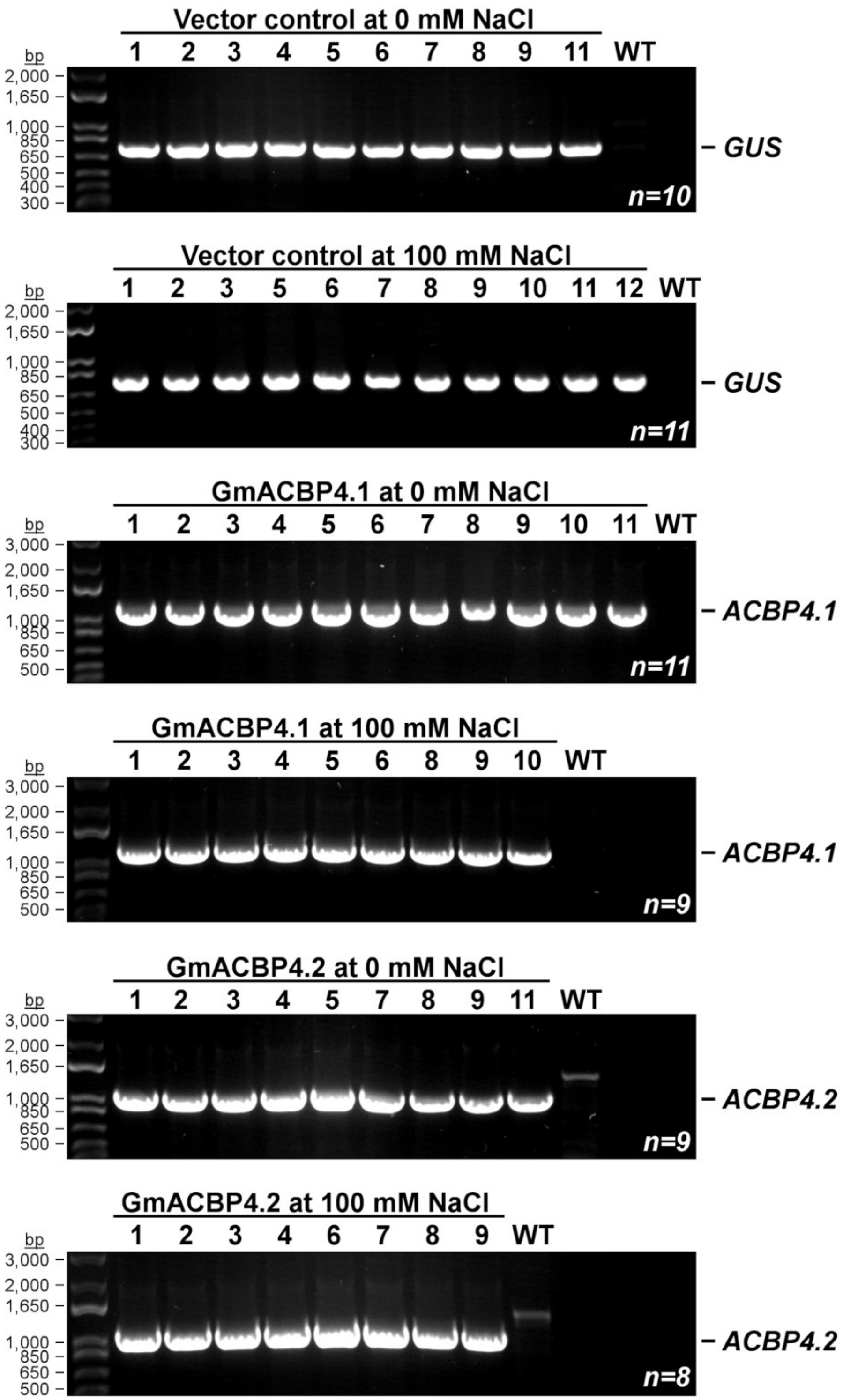

Supplemental Figure 8. PCR verification of transgenic soybean hairy roots. (Supports Figures 7, 9A to 9C)

Transgenic soybean hairy roots were grown with or without 100 mM NaCl for salt tolerance test. The presence of the transgene in hairy roots was verified by PCR. Vector controls were verified by the presence of a *β-GLUCURONIDASE* (*GUS*) fragment (~760 bp) using primers 35SB and ML2894. Hairy roots expressing GmACBP4.1 were verified by the presence of a *GmACBP4.1* fragment (1.1 kb) using primers 35SB and ML3129. Hairy roots expressing GmACBP4.2 were verified by the presence of a *GmACBP4.2* fragment (1 kb) using primers 35SB and ML3165. Genomic DNA from wild-type (WT) cultivated C08 soybean was used as a template for negative controls.

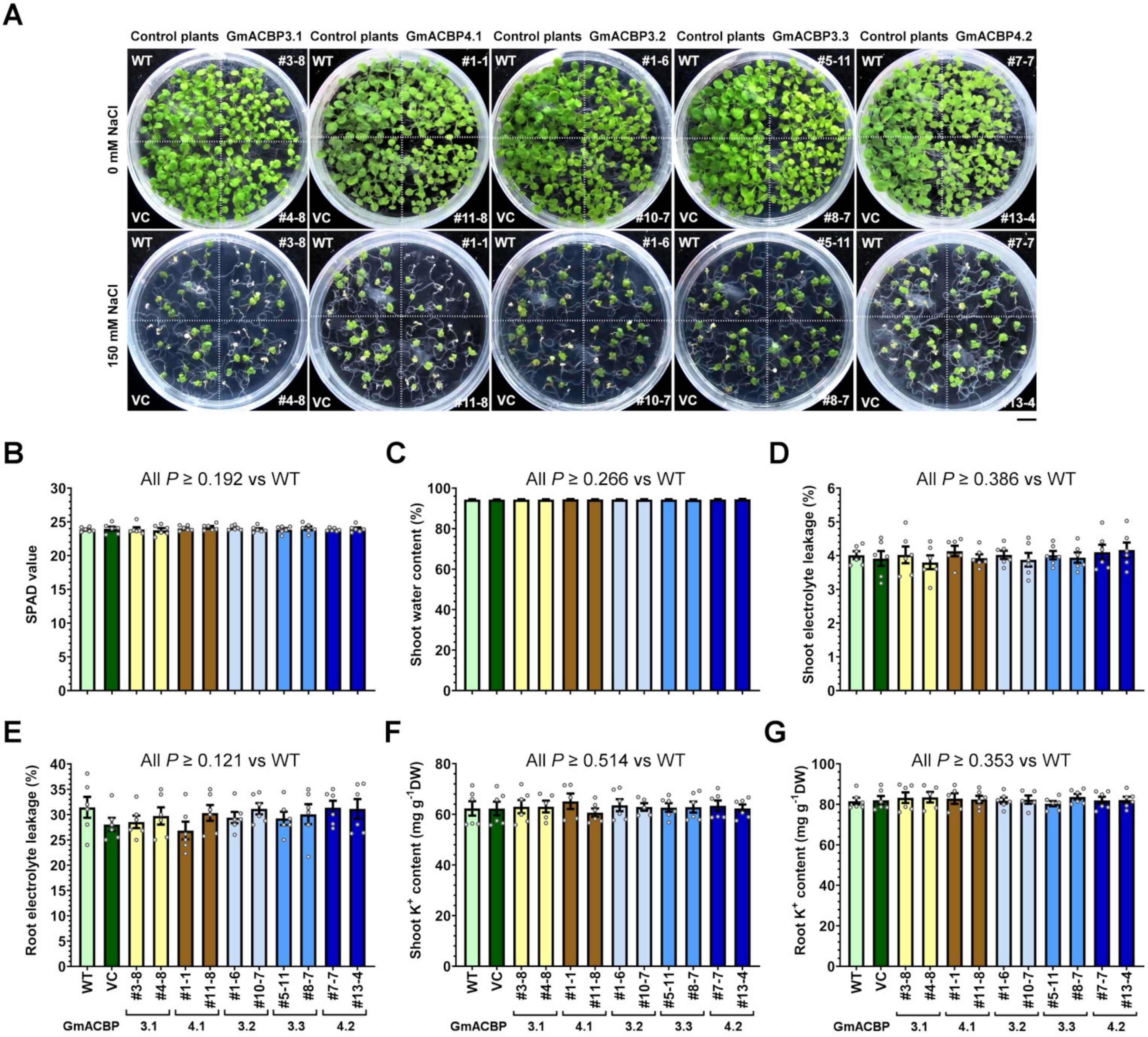

Supplemental Figure 9. Salt tolerance test and mock treatment of transgenic Arabidopsis expressing native form and splice variants of Class II GmACBPs. (Supports Figure 8)

Transgenic Arabidopsis expressing the native forms (GmACBP3.1 and GmACBP4.1) and splice variants (GmACBP3.2, GmACBP3.3 and GmACBP4.2), the wild type (WT) and vector control (VC) were examined.

(A) High-salinity phenotypes of Arabidopsis seedlings. Four-d-old seedlings were transferred to MS plates with or without 150 mM NaCl. Representative photos were taken after 14 d. Bar = 1 cm.

(B–G) Mock treatment of hydroponically-grown Arabidopsis. Four-week-old plants were treated in fresh hydroponic solution without 150 mM NaCl for 10 d. Chlorophyll content measured by the soil plant analysis development (SPAD) meter in **(B)**, and shoot and root electrolyte leakage in **(D–E)** are the mean ± S.E.M. of six plants. Shoot water content in **(C)** is the mean ± S.E.M. of eight to nine plants. Shoot and root K^+ content in **(F–G)** is the mean ± S.E.M. of five to six plants. All genotypes exhibited no statistically significant (*P*>0.05) difference from WT by Student's *t* test (Supplemental Data Set 1).

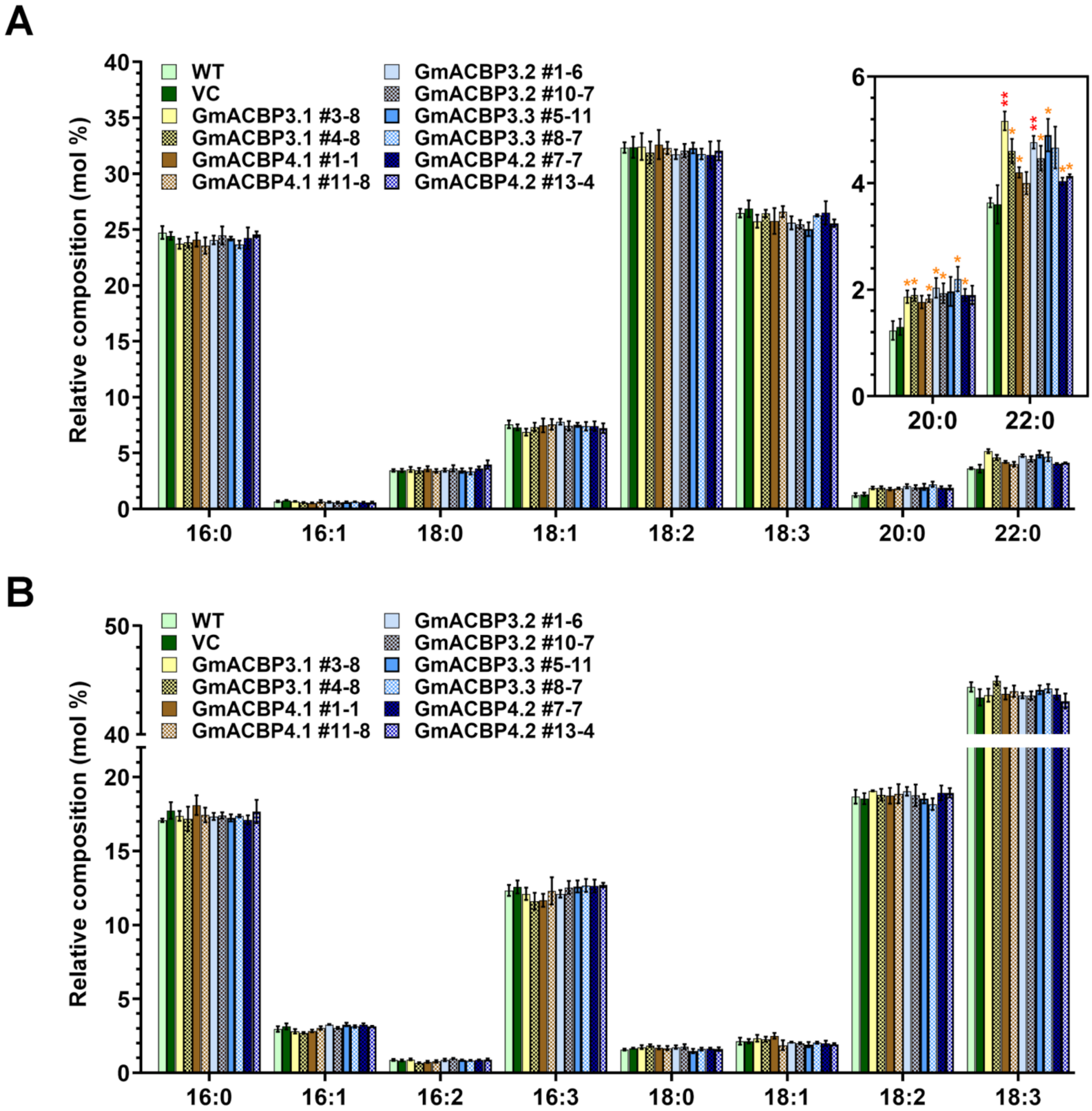

Supplemental Figure 10. Fatty acid composition of transgenic Arabidopsis expressing Class II GmACBPs. (Supports Figure 9A) Fatty acid composition of transgenic Arabidopsis expressing Class II GmACBPs, the wild type (WT) and vector control (VC) was analyzed. Each bar represents the mean of three plants ± S.E.M.

(A) Fatty acid composition of roots. Asterisks indicate statistically significant (*, *P*<0.05; **, *P*<0.01) difference from WT by Student's *t* test (Supplemental Data Set 1).

(B) Fatty acid composition of shoots. All genotypes exhibited no statistically significant (*P*>0.05) difference from WT by Student's *t* test (Supplemental Data Set 1).

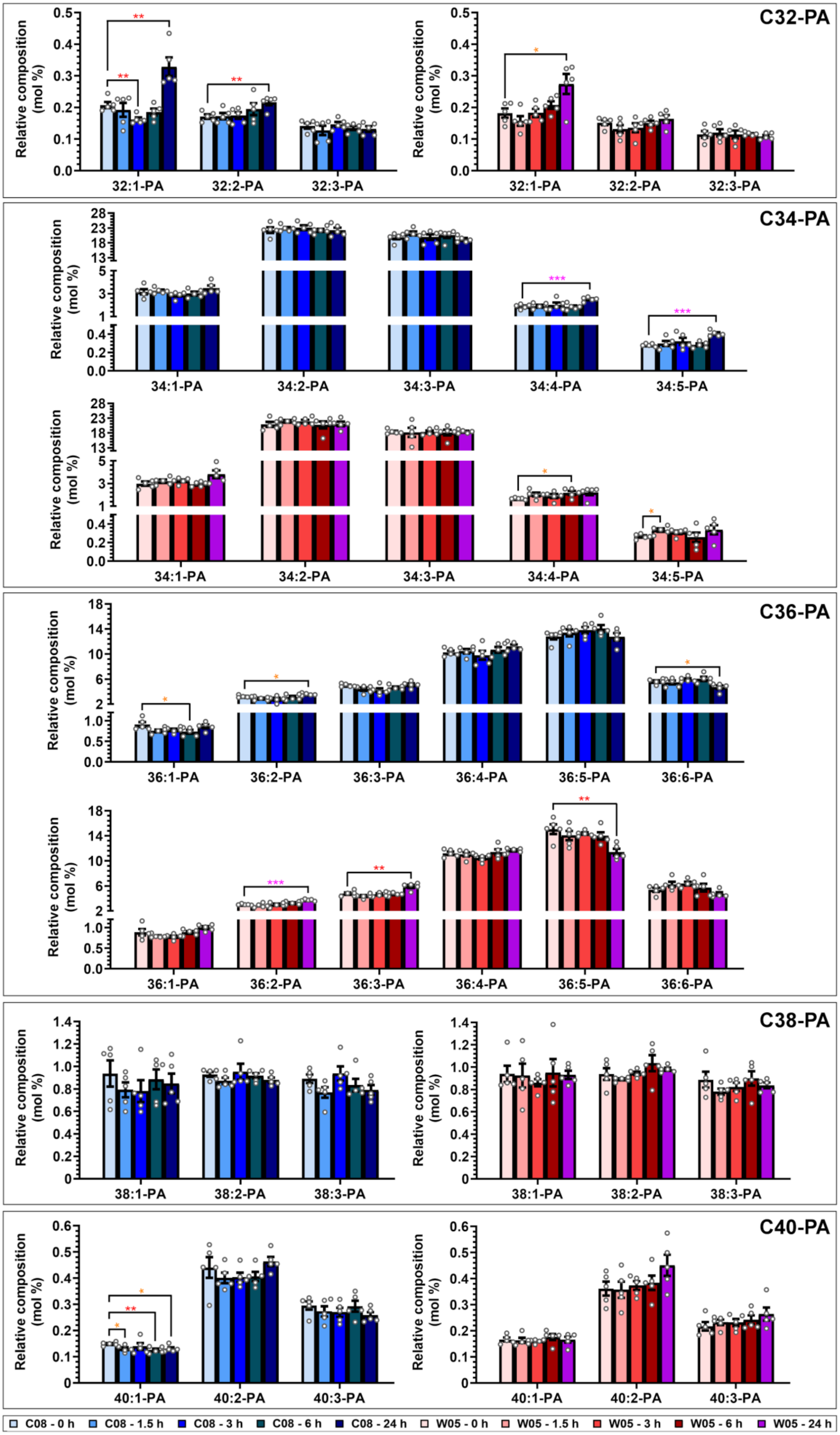
C32-PA
Relative composition (mol %)
32:1-PA
32:2-PA
32:3-PA
C34-PA
34:1-PA
34:2-PA
34:3-PA
34:4-PA
34:5-PA
C36-PA
36:1-PA
36:2-PA
36:3-PA
36:4-PA
36:5-PA
36:6-PA
C38-PA
38:1-PA
38:2-PA
38:3-PA
C40-PA
40:1-PA
40:2-PA
40:3-PA
C08 - 0 h
C08 - 1.5 h
C08 - 3 h
C08 - 6 h
C08 - 24 h
W05 - 0 h
W05 - 1.5 h
W05 - 3 h
W05 - 6 h
W05 - 24 h

Supplemental Figure 11. Molecular species composition of phosphatidic acid in soybean roots under salinity. (Supports Figure 9I)

Molecular species composition of phosphatidic acid (PA) was analyzed after treatment of cultivated C08 and wild W05 soybean roots in hydroponic solution with 0.9% (w/v) NaCl for 0, 1.5, 3, 6 and 24 h. Each bar represents the mean of five plants ± S.E.M. Asterisks indicate statistically significant (*, $P < 0.05$; **, $P < 0.01$; ***, $P < 0.001$) difference from 0 h by Student's *t* test (Supplemental Data Set 1).

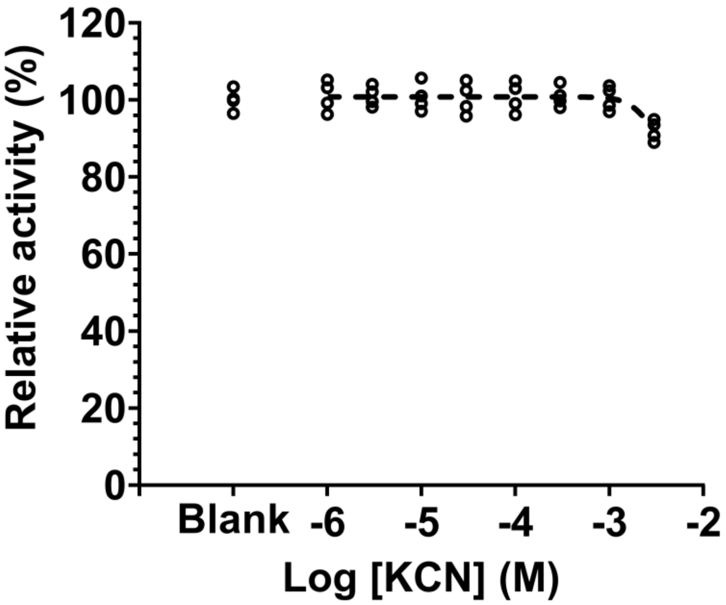

Supplemental Figure 12. *In vitro* enzyme activity of VLXB in the presence of potassium cyanide. (Supports Figures 9C, 9G and 10B)

Purified VLXB:StrepII (100 nM) was preincubated with 0, 1 μM, 3 μM, 10 μM, 30 μM, 100 μM, 300 μM, 1 mM or 3 mM potassium cyanide (KCN) at 25 °C for 10 min, prior to *in vitro* enzyme assays using linoleic acid as a substrate. The activity was normalized to the blank without KCN in the same assay. Data were obtained from four independent experiments.

Supplemental Table 1. Binding constants and thermodynamic parameters for $(His)_6$-GmACBP4.$1_{33–354}$ interaction with acyl-CoAs. (Supports Figure 5A)

Acyl-CoA	*n*	K_D (μM)	ΔH (kcal mol^{-1})	$-T\Delta S$ (kcal mol^{-1})	ΔG (kcal mol^{-1})
C16:0-CoA	1.08 ± 0.02	0.80 ± 0.10	−4.00 ± 0.09	−4.30 ± 0.28	−8.24 ± 0.37
C18:1-CoA	1.22 ± 0.02	0.68 ± 0.08	−4.10 ± 0.08	−4.32 ± 0.42	−8.42 ± 0.50
C18:2-CoA	1.03 ± 0.02	0.92 ± 0.12	−4.58 ± 0.11	−3.64 ± 0.32	−8.22 ± 0.43
C18:3-CoA	1.14 ± 0.03	1.17 ± 0.20	−3.73 ± 0.15	−4.36 ± 0.31	−8.09 ± 0.46

Isothermal titration calorimetry analysis was performed by titration of 250 μL of 15 μM $(His)_6$-GmACBP4.$1_{33–354}$ with 20 injections of 1.8-μL aliquots of 200 μM C16:0-, C18:1-, C18:2- or C18:3-CoAs at 25 ºC. Values are mean ± SD from three independent experiments. *n,* stoichiometry (number of ligand sites per protein); K_D, dissociation constant; ΔH, enthalpy change; $-T\Delta S$, entropy change; ΔG, Gibbs free energy.

Supplemental Table 2. Sequences of primers used in this study.

Name	Nucleotide sequence from 5´ to 3´	Orientation	Use
ML2894	TCTTGCCGTTTTCGTCGGTA	Reverse	Verification of soybean hairy roots transformed with pCambia3301 vector control
ML2999	CTTCCATTTGTGAGTCACTGC	Reverse	qRT-PCR for all *ACBP3* and *ACBP4*
ML3079	ATATTCTAGAATGGCCGAGTGGCAGTC (*Xba*I)	Forward	RT-PCR for *ACBP3* and *ACBP4*; generating pGM943, pGM944, pGM947–pGM952, pGM955–pGM957, pGM960–pGM962, pGM992–pGM996, pGM1008–pGM1015, and pGM1020–pGM1023; creating Y135A, K139A, K161A and Y180A mutants
ML3080	ATATGGATCCCGGGCCATTTACTTCTCCCCCCACAT (*Bam*HI)	Reverse	Generating pGM943, pGM947, and pGM992
ML3081	ATATGGATCCCGGGCCATTTACTTCTTCCCCCACAT (*Bam*HI)	Reverse	Generating pGM944, pGM948, pGM995, pGM1008–pGM1015, and pGM1020–pGM1023; creating Y135A, K139A, K161A and Y180A mutants
ML3128	ATATCTCGAGTCAATTTACTTCTCCCCCCA (*Xho*I)	Reverse	RT-PCR for *ACBP3* and *ACBP4*; generating pGM949, pGM951, and pGM970
ML3129	ATATCTCGAGTCAATTTACTTCTTCCCCCA (*Xho*I)	Reverse	Verification of soybean hairy roots transformed with pGM973; generating pGM950, pGM952, pGM963, pGM971, and pGM973
ML3150	TAGCTCGAGGACGACAATCTCACCGTCAC (*Xho*I)	Forward	Generating pGM958, pGM963, pGM970, and pGM971
ML3151	TAGGAATTCTCAATTTACTTCTCCCCCCAC (*Eco*RI)	Reverse	Generating pGM958
ML3163	ATATCTCGAGTCATCCATCGAGGGCACA (*Xho*I)	Reverse	Generating pGM955
ML3164	ATATCTCGAGTTACGGCAATGCCAATATAGAAATA (*Xho*I)	Reverse	Generating pGM956
ML3165	ATATCTCGAGTTACGGCAATGCCAATACA (*Xho*I)	Reverse	Verification of soybean hairy roots transformed with pGM974; generating pGM957 and pGM974
ML3166	GTGCCAAATGGCAAGCGT	Forward	qRT-PCR for *ACBP3.3*
ML3167	GAGGGCACAAGAAGAAACAGC	Reverse	qRT-PCR for *ACBP3.3*
ML3168	TTCCATGAATTTAAAGGACAGTGAG	Forward	qRT-PCR for *ACBP3.2*
ML3169	AATATAAGTAGTCCCCGACATTAGC	Reverse	qRT-PCR for *ACBP3.2*
ML3170	TTCAATGAATTTAAAGGACAGTGAG	Forward	qRT-PCR for *ACBP4.2*
ML3171	AAGTAGTCCCTGACATTAACACATC	Reverse	qRT-PCR for *ACBP4.2*
ML3238	ATATGGATCCCGGGCCTCCATCGAGGGCACAAGA (*Bam*HI)	Reverse	Generating pGM960 and pGM994
ML3239	ATATGGATCCCGGGCCCGGCAATGCCAATATAGAA (*Bam*HI)	Reverse	Generating pGM961 and pGM993
ML3240	ATATGGATCCCGGGCCCGGCAATGCCAATACAGA (*Bam*HI)	Reverse	Generating pGM962 and pGM996
ML3293	ATATCTCGAGATGGCCGAGTGGCAGTC (*Xho*I)	Forward	Generating pGM973 and pGM974
ML3297	ATATGGATCCGCGCAAATTCTGCTTCATG (*Bam*HI)	Forward	Generating pGM1032
ML3347	GAGGAATAAAAGTGGGGACA	Forward	qRT-PCR for all *ACBP3*
ML3348	GAGGAATAAAAGCGGCGAC	Forward	qRT-PCR for all *ACBP4*
ML3349	TTTTCACTCACTCACTCTGCACT	Forward	RT-PCR and qRT-PCR for *ELF1b*
ML3350	TCCTTTGTCAACTGATCCCCAGAA	Reverse	RT-PCR and qRT-PCR for *ELF1b*
ML3394	ATATTCTAGAATGTTTCCATTCGGGCACA (*Xba*I)	Forward	Generating pGM1002 and pGM1006
ML3395	ATATCTCGAGCGGGCCGATAGAGATACTGTTGGGAATTCC (*Xho*I)	Reverse	Generating pGM1002 and pGM1006

Supplemental Table 2. Sequences of primers used in this study (continued).

Name	Nucleotide sequence from 5´ to 3´	Orientation	Use
ML3400	ATATTCTAGAATGTTCGGAGAACTTAGGGATC (*Xba*I)	Forward	Generating pGM1003 and pAT1007
ML3401	ATATCTCGAGCGGGCCGATAGAGACGCTATTTGGAATTCC (*Xho*I)	Reverse	Generating pGM1003 and pAT1007
ML3413	GACGTGCAGCTACAGCTT**GCG**GGTCTTTACAAGATTGCC	Forward	Creating Y135A mutants
ML3414	GGCAATCTTGTAAAGACC**CGC**AAGCTGTAGCTGCACGTC	Reverse	Creating Y135A mutants
ML3415	GCTTTACGGTCTTTAC**GC**GATTGCCACCGAAGGTC	Forward	Creating K139A mutants
ML3416	GACCTTCGGTGGCAATC**GC**GTAAAGACCGTAAAGC	Reverse	Creating K139A mutants
ML3417	CTCAAAATGACCGCACGTGCC**GCT**TGGCAAGCGTGGCAGAAATTG	Forward	Creating K161A mutants
ML3418	CAATTTCTGCCACGCTTGCCA**AGC**GGCACGTGCGGTCATTTTGAG	Reverse	Creating K161A mutants
ML3419	GAAGATGCAATGCAGAAG**GC**CATTGATATTGTGACCGAG	Forward	Creating Y180A mutants
ML3420	CTCGGTCACAATATCAATG**GC**CTTCTGCATTGCATCTTC	Reverse	Creating Y180A mutants
ML3423	ATATCCCGGGGTTTTCCATTCGGGCACAAG (*Sma*I)	Forward	Generating pGM1024
ML3424	ATATCCCGGGTTAGATAGAGATACTGTTGGGAATTCCT (*Sma*I)	Reverse	Generating pGM1024
ML3425	ATATCCCGGGGTTTCGGAGAACTTAGGGATCTG (*Sma*I)	Forward	Generating pAT1025
ML3426	ATATCCCGGGTCAGATAGAGACGCTATTTGGAATTCC (*Sma*I)	Reverse	Generating pAT1025
ML3432	ATATCTCGAGTTTCCATTCGGGCACAAG (*Xho*I)	Forward	Generating pGM1027
ML3433	ATCTCGAGTTATTTTTCAAATTGAGGATGAGACCAGATAGAGATACTGTTGGGAATTCC (*Xho*I)	Reverse	Generating pGM1027
ML3436	ATATAAGCTTCTATTTTTCAAATTGAGGATGAGACCAAGTAGTGAGGATAGGGGGGA (*Hin*dIII)	Reverse	Generating pGM1032
35SB	CAATCCCACTATCCTTCGCAAGACC	Forward	Verification of soybean hairy roots transformed with pCambia3301 vector control, pGM973 and pGM974

Restriction sites used for subcloning (if any) are underlined and shown in parentheses;

Codons for site-directed mutagenesis are double-underlined with the altered nucleotides in bold.
